# Supplementary material for: Chinese influence in Africa by integrated regret theory and multi-criteria decision analysis
Source: PLoS One. 2025 Nov 12;20(11):e0336681. doi: 10.1371/journal.pone.0336681 (PMC12611122; doi:10.1371/journal.pone.0336681)
Supplement: S1 Data — (DOCX) [file pone.0336681.s001.docx]

**Minimal Data Set**

Table A1. The decision matrix for 2018 data

| **Country** | **C1** | **C2** | **C3** | **C4** | **C5** | **C6** |
| --- | --- | --- | --- | --- | --- | --- |
|  | million USD | million USD | million USD | workers | million USD | million USD |
| DZA | 2062.86 | 7923.38 | 1178.82 | 39106.00 | 7521.46 | 0.00 |
| AGO | 2299.19 | 2234.93 | 25652.02 | 14897.00 | 4543.19 | 1299.00 |
| BEN | 103.99 | 2150.68 | 48.32 | 357.00 | 143.70 | 0.00 |
| BWA | 258.16 | 281.47 | 14.23 | 462.00 | 259.56 | 0.00 |
| BFA | 0.20 | 223.34 | 95.74 | 21.00 | 40.45 | 0.00 |
| BDI | 12.52 | 36.90 | 11.93 | 675.00 | 40.78 | 0.00 |
| CMR | 499.21 | 1706.52 | 1095.69 | 2718.00 | 1252.10 | 164.14 |
| CPV | 14.63 | 78.05 | 0.29 | 393.00 | 27.44 | 0.00 |
| CAF | 88.13 | 18.78 | 54.11 | 185.00 | 22.53 | 0.00 |
| TCD | 592.59 | 185.53 | 93.92 | 1575.00 | 498.33 | 0.00 |
| COM | 5.45 | 79.20 | 0.03 | 129.00 | 29.24 | 85.65 |
| COG | 795.10 | 444.88 | 6702.54 | 3089.00 | 477.05 | 0.00 |
| COD | 4444.46 | 1772.66 | 5682.94 | 6230.00 | 1820.15 | 694.65 |
| CIV | 441.54 | 1890.20 | 254.38 | 2009.00 | 850.48 | 291.66 |
| DJI | 178.49 | 1871.68 | 0.20 | 781.00 | 301.47 | 0.00 |
| EGY | 1079.26 | 12020.88 | 1834.53 | 1171.00 | 2045.60 | 1857.40 |
| GNQ | 552.85 | 145.17 | 2131.56 | 3065.00 | 542.94 | 0.00 |
| ERI | 223.94 | 42.84 | 314.58 | 109.00 | 40.23 | 87.00 |
| ETH | 2568.16 | 2537.68 | 345.31 | 8310.00 | 4001.79 | 243.20 |
| GAB | 258.66 | 386.09 | 2964.33 | 608.00 | 161.04 | 51.75 |
| GMB | 24.79 | 426.52 | 20.06 | 297.00 | 37.17 | 25.00 |
| GHA | 1797.47 | 4821.89 | 2426.14 | 1632.00 | 1131.76 | 665.55 |
| GIN | 742.44 | 1353.33 | 2246.15 | 3606.00 | 819.87 | 2017.00 |
| KEN | 1755.88 | 5204.94 | 174.20 | 8503.00 | 4352.74 | 162.88 |
| LSO | 6.53 | 64.20 | 30.10 | 126.00 | 11.68 | 105.82 |
| LBR | 260.39 | 1941.89 | 89.25 | 805.00 | 196.83 | 0.00 |
| MDG | 803.35 | 1015.76 | 214.71 | 161.00 | 167.40 | 102.63 |
| MWI | 292.10 | 222.35 | 27.36 | 266.00 | 89.02 | 0.00 |
| MLI | 301.47 | 345.61 | 88.51 | 597.00 | 400.72 | 168.85 |
| MRT | 232.61 | 1039.98 | 858.21 | 856.00 | 183.24 |  |
| MUS | 997.66 | 807.78 | 37.46 | 688.00 | 124.18 | 0.00 |
| MAR | 382.29 | 3690.13 | 711.07 | 274.00 | 683.04 | 150.00 |
| MOZ | 1410.17 | 1869.33 | 651.38 | 2329.00 | 804.22 | 0.00 |
| NAM | 426.15 | 319.47 | 502.98 | 1066.00 | 458.99 | 9.07 |
| NER | 758.40 | 114.66 | 171.11 | 713.00 | 171.58 | 0.00 |
| NGA | 2453.49 | 13500.04 | 1858.99 | 7180.00 | 4047.85 | 884.12 |
| RWA | 146.82 | 165.17 | 39.96 | 748.00 | 288.05 | 125.66 |
| SEN | 314.65 | 2145.22 | 128.51 | 2425.00 | 1142.90 | 900.02 |
| SYC | 451.91 | 61.34 | 0.03 | 156.00 | 15.91 | 0.00 |
| SLE | 168.06 | 253.20 | 177.19 | 653.00 | 136.11 | 0.00 |
| ZAF | 6531.68 | 16337.35 | 8550.85 | 551.00 | 1381.42 | 2050.23 |
| SSD | 35.69 | 77.08 | 1588.45 | 375.00 | 460.54 | 248.80 |
| SDN | 1325.07 | 1884.77 | 670.03 | 3154.00 | 500.46 | 30.21 |
| TZA | 1302.75 | 3590.57 | 393.92 | 3199.00 | 1076.00 | 0.00 |
| TGO | 102.07 | 1991.48 | 145.76 | 376.00 | 225.18 | 0.00 |
| TUN | 21.53 | 1415.92 | 194.96 | 243.00 | 111.51 | 0.00 |
| UGA | 798.17 | 706.40 | 47.17 | 3913.00 | 2147.67 | 0.00 |
| ZMB | 3523.02 | 969.45 | 4132.38 | 5544.00 | 2703.45 | 1037.58 |
| ZWE | 1766.25 | 446.07 | 890.33 | 589.00 | 320.33 | 162.86 |

Table A2. The decision matrix for 2019 data

| **Country** | **C1** | **C2** | **C3** | **C4** | **C5** | **C6** |
| --- | --- | --- | --- | --- | --- | --- |
|  | million USD | million USD | million USD | workers | million USD | million USD |
| DZA | 1775.35 | 6945.57 | 1141.66 | 28961.00 | 6335.48 | 0.00 |
| AGO | 2890.73 | 2057.49 | 23308.30 | 10725.00 | 2865.88 | 106.43 |
| BEN | 91.44 | 2155.75 | 154.36 | 987.00 | 244.18 | 159.26 |
| BWA | 186.28 | 302.21 | 17.13 | 392.00 | 168.28 | 0.00 |
| BFA | 1.49 | 261.49 | 59.96 | 3.00 | 13.54 | 77.35 |
| BDI | 8.20 | 66.39 | 12.94 | 598.00 | 86.19 | 0.00 |
| CMR | 303.90 | 1676.31 | 1015.04 | 2398.00 | 1399.05 | 97.42 |
| CPV | 2.34 | 63.42 | 0.03 | 188.00 | 66.71 | 0.00 |
| CAF | 13.98 | 25.66 | 36.20 | 329.00 | 87.43 | 29.01 |
| TCD | 648.52 | 278.77 | 439.83 | 2271.00 | 732.83 | 188.43 |
| COM | 1.83 | 73.68 | 0.03 | 116.00 | 32.47 | 0.00 |
| COG | 609.84 | 434.93 | 5934.47 | 2620.00 | 369.12 | 0.00 |
| COD | 5596.60 | 2077.31 | 4436.61 | 4806.00 | 2446.50 | 0.00 |
| CIV | 564.34 | 2044.09 | 460.76 | 2179.00 | 1092.77 | 999.29 |
| DJI | 125.26 | 2210.29 | 20.14 | 413.00 | 229.01 | 0.00 |
| EGY | 1085.80 | 12217.15 | 996.82 | 1663.00 | 3189.59 | 3750.00 |
| GNQ | 404.13 | 113.30 | 1723.01 | 2140.00 | 581.97 | 281.35 |
| ERI | 223.29 | 48.61 | 209.89 | 166.00 | 2.56 | 0.00 |
| ETH | 2558.87 | 2322.76 | 343.91 | 7073.00 | 2495.97 | 500.00 |
| GAB | 252.21 | 377.22 | 4612.51 | 746.00 | 271.01 | 137.81 |
| GMB | 13.90 | 512.67 | 71.44 | 141.00 | 74.32 | 0.00 |
| GHA | 1831.29 | 4906.97 | 2543.92 | 1502.00 | 817.03 | 695.13 |
| GIN | 763.26 | 1718.20 | 2504.86 | 3937.00 | 1218.00 | 0.00 |
| KEN | 1624.23 | 4984.58 | 181.21 | 7596.00 | 4167.63 | 264.66 |
| LSO | 5.93 | 72.06 | 31.27 | 188.00 | 71.70 | 0.00 |
| LBR | 167.65 | 3909.85 | 112.42 | 499.00 | 440.20 | 0.00 |
| MDG | 272.91 | 1073.08 | 204.56 | 229.00 | 114.82 | 157.74 |
| MWI | 161.45 | 260.10 | 15.71 | 245.00 | 153.74 | 0.00 |
| MLI | 305.00 | 435.55 | 160.64 | 885.00 | 425.72 | 0.00 |
| MRT | 181.40 | 1026.41 | 915.85 | 967.00 | 295.04 | 87.00 |
| MUS | 1291.68 | 804.58 | 38.86 | 540.00 | 118.15 | 0.00 |
| MAR | 303.29 | 4030.06 | 635.51 | 414.00 | 335.25 | 0.00 |
| MOZ | 1146.75 | 1956.81 | 711.72 | 2064.00 | 1001.44 | 0.00 |
| NAM | 363.59 | 200.89 | 511.61 | 722.00 | 445.71 | 0.00 |
| NER | 956.71 | 288.82 | 223.87 | 838.00 | 299.65 | 0.00 |
| NGA | 2194.00 | 16634.07 | 2652.14 | 7720.00 | 4595.59 | 392.53 |
| RWA | 167.51 | 264.79 | 35.06 | 858.00 | 308.73 | 0.00 |
| SEN | 234.24 | 2212.62 | 300.98 | 1912.00 | 884.16 | 0.00 |
| SYC | 414.05 | 65.31 | 0.07 | 166.00 | 26.00 | 0.00 |
| SLE | 165.32 | 313.69 | 197.45 | 575.00 | 128.31 | 30.00 |
| ZAF | 6146.57 | 16560.93 | 9595.42 | 598.00 | 557.88 | 0.00 |
| SSD | 26.88 | 122.63 | 1510.94 | 677.00 | 242.91 | 0.00 |
| SDN | 1203.09 | 2292.12 | 740.42 | 3078.00 | 381.28 | 0.00 |
| TZA | 1335.54 | 3814.73 | 366.34 | 4222.00 | 1282.83 | 0.00 |
| TGO | 101.16 | 2131.52 | 180.43 | 116.00 | 119.41 | 0.00 |
| TUN | 36.62 | 1365.19 | 208.77 | 232.00 | 93.67 | 0.00 |
| UGA | 669.94 | 741.31 | 42.28 | 3608.00 | 1540.38 | 0.00 |
| ZMB | 2863.79 | 970.30 | 3298.46 | 6114.00 | 2574.97 | 101.48 |
| ZWE | 1771.48 | 368.35 | 974.03 | 871.00 | 555.69 | 71.00 |

Table A3. The decision matrix for 2020 data

| **Country** | **C1** | **C2** | **C3** | **C4** | **C5** | **C6** |
| --- | --- | --- | --- | --- | --- | --- |
|  | million USD | million USD | million USD | workers | million USD | million USD |
| DZA | 1643.52 | 5597.42 | 996.57 | 13082.00 | 4688.11 | 0.00 |
| AGO | 2690.09 | 1747.85 | 14513.45 | 5115.00 | 1514.73 | 0.00 |
| BEN | 75.46 | 988.87 | 58.25 | 789.00 | 234.31 | 0.00 |
| BWA | 190.43 | 235.69 | 87.49 | 201.00 | 193.91 | 0.00 |
| BFA | 1.71 | 322.52 | 77.69 | 0.00 | 0.00 | 80.00 |
| BDI | 10.52 | 76.10 | 7.67 | 232.00 | 50.46 | 0.00 |
| CMR | 442.74 | 2022.78 | 751.65 | 2315.00 | 892.51 | 0.00 |
| CPV | 2.82 | 77.79 | 1.21 | 97.00 | 26.00 | 0.00 |
| CAF | 14.99 | 27.96 | 57.07 | 194.00 | 49.95 | 0.00 |
| TCD | 654.81 | 298.32 | 420.91 | 1656.00 | 537.09 | 0.00 |
| COM | 1.18 | 50.54 | 0.12 | 118.00 | 38.59 | 0.00 |
| COG | 1130.88 | 601.52 | 3357.05 | 1295.00 | 573.38 | 0.00 |
| COD | 3688.13 | 2013.12 | 7030.64 | 5209.00 | 2025.03 | 150.00 |
| CIV | 666.85 | 2333.50 | 576.14 | 1291.00 | 917.50 | 59.89 |
| DJI | 98.83 | 2308.24 | 46.25 | 542.00 | 121.74 | 0.00 |
| EGY | 1191.72 | 13623.26 | 905.85 | 1546.00 | 2993.39 | 0.00 |
| GNQ | 336.01 | 122.10 | 1154.28 | 956.00 | 340.48 | 0.00 |
| ERI | 199.86 | 70.49 | 289.36 | 160.00 | 21.64 | 0.00 |
| ETH | 2992.80 | 2233.34 | 338.42 | 7540.00 | 2924.73 | 0.00 |
| GAB | 259.19 | 415.58 | 3217.54 | 536.00 | 68.31 | 0.00 |
| GMB | 19.03 | 535.90 | 29.74 | 196.00 | 55.70 | 0.00 |
| GHA | 1584.03 | 6756.06 | 1740.92 | 1903.00 | 1122.19 | 498.98 |
| GIN | 472.82 | 1912.38 | 2475.69 | 4847.00 | 1777.94 | 0.00 |
| KEN | 2154.30 | 5410.77 | 151.00 | 3936.00 | 2957.00 | 0.00 |
| LSO | 17.58 | 59.38 | 12.26 | 156.00 | 79.27 | 66.83 |
| LBR | 168.88 | 3404.58 | 87.99 | 282.00 | 227.27 | 0.00 |
| MDG | 390.74 | 997.51 | 138.70 | 254.00 | 199.54 | 48.99 |
| MWI | 173.16 | 218.59 | 12.21 | 421.00 | 165.98 | 100.43 |
| MLI | 308.21 | 468.41 | 168.90 | 700.00 | 282.04 | 0.00 |
| MRT | 182.95 | 740.16 | 1227.81 | 559.00 | 138.82 | 0.00 |
| MUS | 886.71 | 699.89 | 26.35 | 365.00 | 186.91 | 0.00 |
| MAR | 383.47 | 4172.59 | 591.09 | 410.00 | 219.84 | 0.00 |
| MOZ | 1317.49 | 1999.92 | 577.17 | 2107.00 | 675.68 | 132.00 |
| NAM | 354.89 | 223.11 | 557.87 | 495.00 | 278.91 | 0.00 |
| NER | 1176.62 | 303.06 | 224.70 | 1244.00 | 400.47 | 0.00 |
| NGA | 2367.54 | 16776.74 | 2453.39 | 6325.00 | 3510.36 | 0.00 |
| RWA | 170.80 | 282.54 | 38.18 | 753.00 | 317.70 | 214.00 |
| SEN | 426.76 | 2563.36 | 315.24 | 905.00 | 521.07 | 0.00 |
| SYC | 439.51 | 57.66 | 0.04 | 138.00 | 13.93 | 0.00 |
| SLE | 134.17 | 371.37 | 158.55 | 252.00 | 92.08 | 0.00 |
| ZAF | 5417.22 | 15243.39 | 9793.79 | 181.00 | 491.27 | 0.00 |
| SSD | 25.60 | 156.05 | 676.87 | 701.00 | 294.61 | 0.00 |
| SDN | 1120.30 | 2511.65 | 766.26 | 1196.00 | 317.21 | 0.00 |
| TZA | 1541.00 | 4174.64 | 409.04 | 2884.00 | 1456.23 | 0.00 |
| TGO | 99.45 | 2461.12 | 164.20 | 154.00 | 84.97 | 0.00 |
| TUN | 29.09 | 1427.65 | 222.66 | 162.00 | 85.22 | 0.00 |
| UGA | 711.96 | 791.65 | 39.69 | 1821.00 | 1219.40 | 297.74 |
| ZMB | 3055.00 | 681.36 | 3306.18 | 4185.00 | 2022.19 | 99.89 |
| ZWE | 1795.80 | 524.67 | 873.95 | 1033.00 | 729.61 | 0.00 |

Table A4. The decision matrix for 2021 data

| **Country** | **C1** | **C2** | **C3** | **C4** | **C5** | **C6** |
| --- | --- | --- | --- | --- | --- | --- |
|  | *million USD* | *million USD* | *million USD* | *workers* | *million USD* | *million USD* |
| DZA | 1716.02 | 6349.68 | 1080.77 | 7951.00 | 3824.22 | 0.00 |
| AGO | 2710.09 | 2491.55 | 20908.23 | 4445.00 | 2099.46 | 110.03 |
| BEN | 84.71 | 1227.52 | 231.57 | 595.00 | 271.16 | 670.74 |
| BWA | 153.52 | 255.50 | 172.62 | 263.00 | 395.41 | 0.00 |
| BFA | 7.05 | 442.69 | 188.45 | 59.00 | 32.52 | 0.00 |
| BDI | 16.91 | 126.90 | 8.61 | 280.00 | 59.41 | 0.00 |
| CMR | 433.55 | 2714.02 | 1636.69 | 2058.00 | 853.44 | 0.00 |
| CPV | 1.31 | 84.54 | 1.15 | 20.00 | 19.83 | 0.00 |
| CAF | 11.74 | 45.53 | 36.07 | 198.00 | 72.26 | 0.00 |
| TCD | 611.32 | 365.27 | 196.80 | 1354.00 | 748.12 | 0.00 |
| COM | 1.04 | 56.95 | 0.11 | 72.00 | 37.63 | 0.00 |
| COG | 967.09 | 680.35 | 4673.95 | 1077.00 | 623.24 | 0.00 |
| COD | 4259.36 | 259.61 | 11671.20 | 5425.00 | 2070.05 | 0.00 |
| CIV | 793.19 | 3120.94 | 641.89 | 1818.00 | 1066.57 | 0.00 |
| DJI | 82.43 | 2572.27 | 55.81 | 390.00 | 160.55 | 0.00 |
| EGY | 1273.44 | 18264.73 | 1704.41 | 4412.00 | 1945.45 | 0.00 |
| GNQ | 280.65 | 123.98 | 1215.14 | 664.00 | 363.75 | 0.00 |
| ERI | 208.17 | 69.96 | 389.73 | 175.00 | 3.81 | 0.00 |
| ETH | 2810.90 | 2290.95 | 366.01 | 5275.00 | 2019.84 | 0.00 |
| GAB | 218.31 | 436.24 | 2584.61 | 229.00 | 216.56 | 0.00 |
| GMB | 19.38 | 543.95 | 44.60 | 68.00 | 16.40 | 0.00 |
| GHA | 1093.54 | 8104.18 | 1466.78 | 1420.00 | 1584.13 | 0.00 |
| GIN | 959.33 | 2158.47 | 2792.45 | 4674.00 | 1100.59 | 0.00 |
| KEN | 2259.81 | 6732.05 | 226.54 | 3599.00 | 2421.20 | 0.00 |
| LSO | 14.51 | 84.44 | 23.66 | 237.00 | 106.22 | 0.00 |
| LBR | 162.78 | 5687.52 | 23.73 | 227.00 | 206.62 | 0.00 |
| MDG | 322.87 | 1283.18 | 331.90 | 890.00 | 368.65 | 0.00 |
| MWI | 176.72 | 280.53 | 9.81 | 378.00 | 174.94 | 0.00 |
| MLI | 438.61 | 594.89 | 127.55 | 381.00 | 442.19 | 0.00 |
| MRT | 142.77 | 909.96 | 1793.73 | 504.00 | 46.68 | 0.00 |
| MUS | 1058.82 | 877.78 | 35.74 | 484.00 | 121.13 | 0.00 |
| MAR | 349.20 | 5689.73 | 824.65 | 325.00 | 223.61 | 0.00 |
| MOZ | 1263.60 | 2895.61 | 1141.13 | 1465.00 | 770.74 | 0.00 |
| NAM | 220.51 | 401.67 | 734.60 | 399.00 | 290.48 | 0.00 |
| NER | 1423.90 | 391.59 | 369.38 | 971.00 | 639.17 | 0.00 |
| NGA | 2695.79 | 22636.16 | 3034.65 | 5636.00 | 4113.01 | 0.00 |
| RWA | 203.10 | 335.14 | 28.72 | 727.00 | 334.21 | 0.00 |
| SEN | 438.85 | 3353.69 | 439.52 | 1386.00 | 824.85 | 442.11 |
| SYC | 492.69 | 68.65 | 0.01 | 94.00 | 10.98 | 0.00 |
| SLE | 106.44 | 489.12 | 403.42 | 194.00 | 99.61 | 0.00 |
| ZAF | 5294.17 | 21115.30 | 13571.17 | 254.00 | 805.39 | 0.00 |
| SSD | 80.72 | 139.82 | 299.41 | 641.00 | 233.38 | 0.00 |
| SDN | 1115.52 | 1816.39 | 779.80 | 1088.00 | 350.75 | 0.00 |
| TZA | 1577.07 | 6137.12 | 604.69 | 2964.00 | 1666.00 | 0.00 |
| TGO | 67.28 | 2952.72 | 532.88 | 235.00 | 95.53 | 0.00 |
| TUN | 33.47 | 1865.63 | 282.72 | 139.00 | 120.75 | 0.00 |
| UGA | 633.12 | 1022.95 | 43.90 | 1848.00 | 1054.30 | 456.37 |
| ZMB | 3029.57 | 780.85 | 4385.25 | 3338.00 | 1249.99 | 0.00 |
| ZWE | 1707.51 | 920.01 | 960.12 | 1049.00 | 698.95 | 0.00 |

Table A5. The normalized decision matrix for 2018 data

| **Country** | **C1** | **C2** | **C3** | **C4** | **C5** | **C6** |
| --- | --- | --- | --- | --- | --- | --- |
| DZA | 0.316 | 0.484 | 0.046 | 1.000 | 1.000 | 0.000 |
| AGO | 0.352 | 0.136 | 1.000 | 0.381 | 0.603 | 0.634 |
| BEN | 0.016 | 0.131 | 0.002 | 0.009 | 0.018 | 0.000 |
| BWA | 0.039 | 0.016 | 0.001 | 0.011 | 0.033 | 0.000 |
| BFA | 0.000 | 0.013 | 0.004 | 0.000 | 0.004 | 0.000 |
| BDI | 0.002 | 0.001 | 0.000 | 0.017 | 0.004 | 0.000 |
| CMR | 0.076 | 0.103 | 0.043 | 0.069 | 0.165 | 0.080 |
| CPV | 0.002 | 0.004 | 0.000 | 0.010 | 0.002 | 0.000 |
| CAF | 0.013 | 0.000 | 0.002 | 0.004 | 0.001 | 0.000 |
| TCD | 0.091 | 0.010 | 0.004 | 0.040 | 0.065 | 0.000 |
| COM | 0.001 | 0.004 | 0.000 | 0.003 | 0.002 | 0.042 |
| COG | 0.122 | 0.026 | 0.261 | 0.078 | 0.062 | 0.000 |
| COD | 0.680 | 0.107 | 0.222 | 0.159 | 0.241 | 0.339 |
| CIV | 0.068 | 0.115 | 0.010 | 0.051 | 0.112 | 0.142 |
| DJI | 0.027 | 0.114 | 0.000 | 0.019 | 0.039 | 0.000 |
| EGY | 0.165 | 0.735 | 0.072 | 0.029 | 0.271 | 0.906 |
| GNQ | 0.085 | 0.008 | 0.083 | 0.078 | 0.071 | 0.000 |
| ERI | 0.034 | 0.001 | 0.012 | 0.002 | 0.004 | 0.042 |
| ETH | 0.393 | 0.154 | 0.013 | 0.212 | 0.531 | 0.119 |
| GAB | 0.040 | 0.023 | 0.116 | 0.015 | 0.020 | 0.025 |
| GMB | 0.004 | 0.025 | 0.001 | 0.007 | 0.003 | 0.012 |
| GHA | 0.275 | 0.294 | 0.095 | 0.041 | 0.149 | 0.325 |
| GIN | 0.114 | 0.082 | 0.088 | 0.092 | 0.108 | 0.984 |
| KEN | 0.269 | 0.318 | 0.007 | 0.217 | 0.578 | 0.079 |
| LSO | 0.001 | 0.003 | 0.001 | 0.003 | 0.000 | 0.052 |
| LBR | 0.040 | 0.118 | 0.003 | 0.020 | 0.025 | 0.000 |
| MDG | 0.123 | 0.061 | 0.008 | 0.004 | 0.021 | 0.050 |
| MWI | 0.045 | 0.012 | 0.001 | 0.006 | 0.010 | 0.000 |
| MLI | 0.046 | 0.020 | 0.003 | 0.015 | 0.052 | 0.082 |
| MRT | 0.036 | 0.063 | 0.033 | 0.021 | 0.023 | 0.000 |
| MUS | 0.153 | 0.048 | 0.001 | 0.017 | 0.015 | 0.000 |
| MAR | 0.058 | 0.225 | 0.028 | 0.006 | 0.089 | 0.073 |
| MOZ | 0.216 | 0.113 | 0.025 | 0.059 | 0.106 | 0.000 |
| NAM | 0.065 | 0.018 | 0.020 | 0.027 | 0.060 | 0.004 |
| NER | 0.116 | 0.006 | 0.007 | 0.018 | 0.021 | 0.000 |
| NGA | 0.376 | 0.826 | 0.072 | 0.183 | 0.537 | 0.431 |
| RWA | 0.022 | 0.009 | 0.002 | 0.019 | 0.037 | 0.061 |
| SEN | 0.048 | 0.130 | 0.005 | 0.062 | 0.151 | 0.439 |
| SYC | 0.069 | 0.003 | 0.000 | 0.003 | 0.001 | 0.000 |
| SLE | 0.026 | 0.014 | 0.007 | 0.016 | 0.017 | 0.000 |
| ZAF | 1.000 | 1.000 | 0.333 | 0.014 | 0.182 | 1.000 |
| SSD | 0.005 | 0.004 | 0.062 | 0.009 | 0.060 | 0.121 |
| SDN | 0.203 | 0.114 | 0.026 | 0.080 | 0.065 | 0.015 |
| TZA | 0.199 | 0.219 | 0.015 | 0.081 | 0.142 | 0.000 |
| TGO | 0.016 | 0.121 | 0.006 | 0.009 | 0.028 | 0.000 |
| TUN | 0.003 | 0.086 | 0.008 | 0.006 | 0.013 | 0.000 |
| UGA | 0.122 | 0.042 | 0.002 | 0.100 | 0.284 | 0.000 |
| ZMB | 0.539 | 0.058 | 0.161 | 0.141 | 0.358 | 0.506 |
| ZWE | 0.270 | 0.026 | 0.035 | 0.015 | 0.041 | 0.079 |

Table A6. The normalized decision matrix for 2019 data

| **Country** | **C1** | **C2** | **C3** | **C4** | **C5** | **C6** |
| --- | --- | --- | --- | --- | --- | --- |
| DZA | 0.289 | 0.417 | 0.049 | 1.000 | 1.000 | 0.000 |
| AGO | 0.470 | 0.122 | 1.000 | 0.370 | 0.452 | 0.028 |
| BEN | 0.015 | 0.128 | 0.007 | 0.034 | 0.038 | 0.042 |
| BWA | 0.030 | 0.017 | 0.001 | 0.013 | 0.026 | 0.000 |
| BFA | 0.000 | 0.014 | 0.003 | 0.000 | 0.002 | 0.021 |
| BDI | 0.001 | 0.002 | 0.001 | 0.021 | 0.013 | 0.000 |
| CMR | 0.049 | 0.099 | 0.044 | 0.083 | 0.221 | 0.026 |
| CPV | 0.000 | 0.002 | 0.000 | 0.006 | 0.010 | 0.000 |
| CAF | 0.002 | 0.000 | 0.002 | 0.011 | 0.013 | 0.008 |
| TCD | 0.105 | 0.015 | 0.019 | 0.078 | 0.115 | 0.050 |
| COM | 0.000 | 0.003 | 0.000 | 0.004 | 0.005 | 0.000 |
| COG | 0.099 | 0.025 | 0.255 | 0.090 | 0.058 | 0.000 |
| COD | 0.911 | 0.124 | 0.190 | 0.166 | 0.386 | 0.000 |
| CIV | 0.092 | 0.122 | 0.020 | 0.075 | 0.172 | 0.266 |
| DJI | 0.020 | 0.132 | 0.001 | 0.014 | 0.036 | 0.000 |
| EGY | 0.176 | 0.734 | 0.043 | 0.057 | 0.503 | 1.000 |
| GNQ | 0.066 | 0.005 | 0.074 | 0.074 | 0.091 | 0.075 |
| ERI | 0.036 | 0.001 | 0.009 | 0.006 | 0.000 | 0.000 |
| ETH | 0.416 | 0.138 | 0.015 | 0.244 | 0.394 | 0.133 |
| GAB | 0.041 | 0.021 | 0.198 | 0.026 | 0.042 | 0.037 |
| GMB | 0.002 | 0.029 | 0.003 | 0.005 | 0.011 | 0.000 |
| GHA | 0.298 | 0.294 | 0.109 | 0.052 | 0.129 | 0.185 |
| GIN | 0.124 | 0.102 | 0.107 | 0.136 | 0.192 | 0.000 |
| KEN | 0.264 | 0.299 | 0.008 | 0.262 | 0.658 | 0.071 |
| LSO | 0.001 | 0.003 | 0.001 | 0.006 | 0.011 | 0.000 |
| LBR | 0.027 | 0.234 | 0.005 | 0.017 | 0.069 | 0.000 |
| MDG | 0.044 | 0.063 | 0.009 | 0.008 | 0.018 | 0.042 |
| MWI | 0.026 | 0.014 | 0.001 | 0.008 | 0.024 | 0.000 |
| MLI | 0.049 | 0.025 | 0.007 | 0.030 | 0.067 | 0.000 |
| MRT | 0.029 | 0.060 | 0.039 | 0.033 | 0.046 | 0.023 |
| MUS | 0.210 | 0.047 | 0.002 | 0.019 | 0.018 | 0.000 |
| MAR | 0.049 | 0.241 | 0.027 | 0.014 | 0.053 | 0.000 |
| MOZ | 0.186 | 0.116 | 0.031 | 0.071 | 0.158 | 0.000 |
| NAM | 0.059 | 0.011 | 0.022 | 0.025 | 0.070 | 0.000 |
| NER | 0.155 | 0.016 | 0.010 | 0.029 | 0.047 | 0.000 |
| NGA | 0.357 | 1.000 | 0.114 | 0.266 | 0.725 | 0.105 |
| RWA | 0.027 | 0.014 | 0.002 | 0.030 | 0.048 | 0.000 |
| SEN | 0.038 | 0.132 | 0.013 | 0.066 | 0.139 | 0.000 |
| SYC | 0.067 | 0.002 | 0.000 | 0.006 | 0.004 | 0.000 |
| SLE | 0.027 | 0.017 | 0.008 | 0.020 | 0.020 | 0.008 |
| ZAF | 1.000 | 0.996 | 0.412 | 0.021 | 0.088 | 0.000 |
| SSD | 0.004 | 0.006 | 0.065 | 0.023 | 0.038 | 0.000 |
| SDN | 0.196 | 0.136 | 0.032 | 0.106 | 0.060 | 0.000 |
| TZA | 0.217 | 0.228 | 0.016 | 0.146 | 0.202 | 0.000 |
| TGO | 0.016 | 0.127 | 0.008 | 0.004 | 0.018 | 0.000 |
| TUN | 0.006 | 0.081 | 0.009 | 0.008 | 0.014 | 0.000 |
| UGA | 0.109 | 0.043 | 0.002 | 0.124 | 0.243 | 0.000 |
| ZMB | 0.466 | 0.057 | 0.142 | 0.211 | 0.406 | 0.027 |
| ZWE | 0.288 | 0.021 | 0.042 | 0.030 | 0.087 | 0.019 |

Table A7. The normalized decision matrix for 2020 data

| **Country** | **C1** | **C2** | **C3** | **C4** | **C5** | **C6** |
| --- | --- | --- | --- | --- | --- | --- |
| DZA | 0.303 | 0.333 | 0.069 | 1.000 | 1.000 | 0.000 |
| AGO | 0.496 | 0.103 | 1.000 | 0.391 | 0.323 | 0.000 |
| BEN | 0.014 | 0.057 | 0.004 | 0.060 | 0.050 | 0.000 |
| BWA | 0.035 | 0.012 | 0.006 | 0.015 | 0.041 | 0.000 |
| BFA | 0.000 | 0.018 | 0.005 | 0.000 | 0.000 | 0.160 |
| BDI | 0.002 | 0.003 | 0.001 | 0.018 | 0.011 | 0.000 |
| CMR | 0.082 | 0.119 | 0.052 | 0.177 | 0.190 | 0.000 |
| CPV | 0.000 | 0.003 | 0.000 | 0.007 | 0.006 | 0.000 |
| CAF | 0.003 | 0.000 | 0.004 | 0.015 | 0.011 | 0.000 |
| TCD | 0.121 | 0.016 | 0.029 | 0.127 | 0.115 | 0.000 |
| COM | 0.000 | 0.001 | 0.000 | 0.009 | 0.008 | 0.000 |
| COG | 0.209 | 0.034 | 0.231 | 0.099 | 0.122 | 0.000 |
| COD | 0.681 | 0.119 | 0.484 | 0.398 | 0.432 | 0.301 |
| CIV | 0.123 | 0.138 | 0.040 | 0.099 | 0.196 | 0.120 |
| DJI | 0.018 | 0.136 | 0.003 | 0.041 | 0.026 | 0.000 |
| EGY | 0.220 | 0.812 | 0.062 | 0.118 | 0.639 | 0.000 |
| GNQ | 0.062 | 0.006 | 0.080 | 0.073 | 0.073 | 0.000 |
| ERI | 0.037 | 0.003 | 0.020 | 0.012 | 0.005 | 0.000 |
| ETH | 0.552 | 0.132 | 0.023 | 0.576 | 0.624 | 0.000 |
| GAB | 0.048 | 0.023 | 0.222 | 0.041 | 0.015 | 0.000 |
| GMB | 0.003 | 0.030 | 0.002 | 0.015 | 0.012 | 0.000 |
| GHA | 0.292 | 0.402 | 0.120 | 0.145 | 0.239 | 1.000 |
| GIN | 0.087 | 0.113 | 0.171 | 0.371 | 0.379 | 0.000 |
| KEN | 0.398 | 0.321 | 0.010 | 0.301 | 0.631 | 0.000 |
| LSO | 0.003 | 0.002 | 0.001 | 0.012 | 0.017 | 0.134 |
| LBR | 0.031 | 0.202 | 0.006 | 0.022 | 0.048 | 0.000 |
| MDG | 0.072 | 0.058 | 0.010 | 0.019 | 0.043 | 0.098 |
| MWI | 0.032 | 0.011 | 0.001 | 0.032 | 0.035 | 0.201 |
| MLI | 0.057 | 0.026 | 0.012 | 0.054 | 0.060 | 0.000 |
| MRT | 0.034 | 0.043 | 0.085 | 0.043 | 0.030 | 0.000 |
| MUS | 0.164 | 0.040 | 0.002 | 0.028 | 0.040 | 0.000 |
| MAR | 0.071 | 0.247 | 0.041 | 0.031 | 0.047 | 0.000 |
| MOZ | 0.243 | 0.118 | 0.040 | 0.161 | 0.144 | 0.265 |
| NAM | 0.065 | 0.012 | 0.038 | 0.038 | 0.059 | 0.000 |
| NER | 0.217 | 0.016 | 0.015 | 0.095 | 0.085 | 0.000 |
| NGA | 0.437 | 1.000 | 0.169 | 0.483 | 0.749 | 0.000 |
| RWA | 0.031 | 0.015 | 0.003 | 0.058 | 0.068 | 0.429 |
| SEN | 0.079 | 0.151 | 0.022 | 0.069 | 0.111 | 0.000 |
| SYC | 0.081 | 0.002 | 0.000 | 0.011 | 0.003 | 0.000 |
| SLE | 0.025 | 0.021 | 0.011 | 0.019 | 0.020 | 0.000 |
| ZAF | 1.000 | 0.908 | 0.675 | 0.014 | 0.105 | 0.000 |
| SSD | 0.005 | 0.008 | 0.047 | 0.054 | 0.063 | 0.000 |
| SDN | 0.207 | 0.148 | 0.053 | 0.091 | 0.068 | 0.000 |
| TZA | 0.284 | 0.248 | 0.028 | 0.220 | 0.311 | 0.000 |
| TGO | 0.018 | 0.145 | 0.011 | 0.012 | 0.018 | 0.000 |
| TUN | 0.005 | 0.084 | 0.015 | 0.012 | 0.018 | 0.000 |
| UGA | 0.131 | 0.046 | 0.003 | 0.139 | 0.260 | 0.597 |
| ZMB | 0.564 | 0.039 | 0.228 | 0.320 | 0.431 | 0.200 |
| ZWE | 0.331 | 0.030 | 0.060 | 0.079 | 0.156 | 0.000 |

Table A8. The normalized decision matrix for 2021 data

| **Country** | **C1** | **C2** | **C3** | **C4** | **C5** | **C6** |
| --- | --- | --- | --- | --- | --- | --- |
| DZA | 0.324 | 0.279 | 0.052 | 1.000 | 0.930 | 0.000 |
| AGO | 0.512 | 0.108 | 1.000 | 0.558 | 0.510 | 0.164 |
| BEN | 0.016 | 0.052 | 0.011 | 0.073 | 0.065 | 1.000 |
| BWA | 0.029 | 0.009 | 0.008 | 0.031 | 0.095 | 0.000 |
| BFA | 0.001 | 0.018 | 0.009 | 0.005 | 0.007 | 0.000 |
| BDI | 0.003 | 0.004 | 0.000 | 0.033 | 0.014 | 0.000 |
| CMR | 0.082 | 0.118 | 0.078 | 0.257 | 0.207 | 0.000 |
| CPV | 0.000 | 0.002 | 0.000 | 0.000 | 0.004 | 0.000 |
| CAF | 0.002 | 0.000 | 0.002 | 0.022 | 0.017 | 0.000 |
| TCD | 0.115 | 0.014 | 0.009 | 0.168 | 0.181 | 0.000 |
| COM | 0.000 | 0.001 | 0.000 | 0.007 | 0.008 | 0.000 |
| COG | 0.183 | 0.028 | 0.224 | 0.133 | 0.151 | 0.000 |
| COD | 0.804 | 0.009 | 0.558 | 0.682 | 0.503 | 0.000 |
| CIV | 0.150 | 0.136 | 0.031 | 0.227 | 0.259 | 0.000 |
| DJI | 0.015 | 0.112 | 0.003 | 0.047 | 0.038 | 0.000 |
| EGY | 0.240 | 0.806 | 0.082 | 0.554 | 0.473 | 0.000 |
| GNQ | 0.053 | 0.003 | 0.058 | 0.081 | 0.088 | 0.000 |
| ERI | 0.039 | 0.001 | 0.019 | 0.020 | 0.000 | 0.000 |
| ETH | 0.531 | 0.099 | 0.018 | 0.663 | 0.491 | 0.000 |
| GAB | 0.041 | 0.017 | 0.124 | 0.026 | 0.052 | 0.000 |
| GMB | 0.003 | 0.022 | 0.002 | 0.006 | 0.003 | 0.000 |
| GHA | 0.206 | 0.357 | 0.070 | 0.177 | 0.385 | 0.000 |
| GIN | 0.181 | 0.094 | 0.134 | 0.587 | 0.267 | 0.000 |
| KEN | 0.427 | 0.296 | 0.011 | 0.451 | 0.588 | 0.000 |
| LSO | 0.003 | 0.002 | 0.001 | 0.027 | 0.025 | 0.000 |
| LBR | 0.031 | 0.250 | 0.001 | 0.026 | 0.049 | 0.000 |
| MDG | 0.061 | 0.055 | 0.016 | 0.110 | 0.089 | 0.000 |
| MWI | 0.033 | 0.010 | 0.000 | 0.045 | 0.042 | 0.000 |
| MLI | 0.083 | 0.024 | 0.006 | 0.046 | 0.107 | 0.000 |
| MRT | 0.027 | 0.038 | 0.086 | 0.061 | 0.010 | 0.000 |
| MUS | 0.200 | 0.037 | 0.002 | 0.059 | 0.029 | 0.000 |
| MAR | 0.066 | 0.250 | 0.039 | 0.038 | 0.053 | 0.000 |
| MOZ | 0.239 | 0.126 | 0.055 | 0.182 | 0.187 | 0.000 |
| NAM | 0.041 | 0.016 | 0.035 | 0.048 | 0.070 | 0.000 |
| NER | 0.269 | 0.015 | 0.018 | 0.120 | 0.155 | 0.000 |
| NGA | 0.509 | 1.000 | 0.145 | 0.708 | 1.000 | 0.000 |
| RWA | 0.038 | 0.013 | 0.001 | 0.089 | 0.080 | 0.000 |
| SEN | 0.083 | 0.146 | 0.021 | 0.172 | 0.200 | 0.659 |
| SYC | 0.093 | 0.001 | 0.000 | 0.009 | 0.002 | 0.000 |
| SLE | 0.020 | 0.020 | 0.019 | 0.022 | 0.023 | 0.000 |
| ZAF | 1.000 | 0.933 | 0.649 | 0.030 | 0.195 | 0.000 |
| SSD | 0.015 | 0.004 | 0.014 | 0.078 | 0.056 | 0.000 |
| SDN | 0.211 | 0.078 | 0.037 | 0.135 | 0.084 | 0.000 |
| TZA | 0.298 | 0.270 | 0.029 | 0.371 | 0.405 | 0.000 |
| TGO | 0.013 | 0.129 | 0.025 | 0.027 | 0.022 | 0.000 |
| TUN | 0.006 | 0.081 | 0.014 | 0.015 | 0.028 | 0.000 |
| UGA | 0.119 | 0.043 | 0.002 | 0.230 | 0.256 | 0.680 |
| ZMB | 0.572 | 0.033 | 0.210 | 0.418 | 0.303 | 0.000 |
| ZWE | 0.322 | 0.039 | 0.046 | 0.130 | 0.169 | 0.000 |

Table A9. The overall utility matrix for 2018 data

| **Country** | **C1** | **C2** | **C3** | **C4** | **C5** | **C6** |
| --- | --- | --- | --- | --- | --- | --- |
| DZA | 0.152 | 0.376 | 0.000 | 1.000 | 1.000 | 0.000 |
| AGO | 0.201 | 0.000 | 1.000 | 0.240 | 0.527 | 0.565 |
| BEN | 0.000 | 0.000 | 0.000 | 0.000 | 0.000 | 0.000 |
| BWA | 0.000 | 0.000 | 0.000 | 0.000 | 0.000 | 0.000 |
| BFA | 0.000 | 0.000 | 0.000 | 0.000 | 0.000 | 0.000 |
| BDI | 0.000 | 0.000 | 0.000 | 0.000 | 0.000 | 0.000 |
| CMR | 0.000 | 0.000 | 0.000 | 0.000 | 0.000 | 0.000 |
| CPV | 0.000 | 0.000 | 0.000 | 0.000 | 0.000 | 0.000 |
| CAF | 0.000 | 0.000 | 0.000 | 0.000 | 0.000 | 0.000 |
| TCD | 0.000 | 0.000 | 0.000 | 0.000 | 0.000 | 0.000 |
| COM | 0.000 | 0.000 | 0.000 | 0.000 | 0.000 | 0.000 |
| COG | 0.000 | 0.000 | 0.076 | 0.000 | 0.000 | 0.000 |
| COD | 0.623 | 0.000 | 0.019 | 0.000 | 0.047 | 0.183 |
| CIV | 0.000 | 0.000 | 0.000 | 0.000 | 0.000 | 0.000 |
| DJI | 0.000 | 0.000 | 0.000 | 0.000 | 0.000 | 0.000 |
| EGY | 0.000 | 0.689 | 0.000 | 0.000 | 0.089 | 0.891 |
| GNQ | 0.000 | 0.000 | 0.000 | 0.000 | 0.000 | 0.000 |
| ERI | 0.000 | 0.000 | 0.000 | 0.000 | 0.000 | 0.000 |
| ETH | 0.257 | 0.000 | 0.000 | 0.005 | 0.437 | 0.000 |
| GAB | 0.000 | 0.000 | 0.000 | 0.000 | 0.000 | 0.000 |
| GMB | 0.000 | 0.000 | 0.000 | 0.000 | 0.000 | 0.000 |
| GHA | 0.095 | 0.122 | 0.000 | 0.000 | 0.000 | 0.164 |
| GIN | 0.000 | 0.000 | 0.000 | 0.000 | 0.000 | 0.981 |
| KEN | 0.086 | 0.155 | 0.000 | 0.012 | 0.496 | 0.000 |
| LSO | 0.000 | 0.000 | 0.000 | 0.000 | 0.000 | 0.000 |
| LBR | 0.000 | 0.000 | 0.000 | 0.000 | 0.000 | 0.000 |
| MDG | 0.000 | 0.000 | 0.000 | 0.000 | 0.000 | 0.000 |
| MWI | 0.000 | 0.000 | 0.000 | 0.000 | 0.000 | 0.000 |
| MLI | 0.000 | 0.000 | 0.000 | 0.000 | 0.000 | 0.000 |
| MRT | 0.000 | 0.000 | 0.000 | 0.000 | 0.000 | 0.000 |
| MUS | 0.000 | 0.000 | 0.000 | 0.000 | 0.000 | 0.000 |
| MAR | 0.000 | 0.024 | 0.000 | 0.000 | 0.000 | 0.000 |
| MOZ | 0.011 | 0.000 | 0.000 | 0.000 | 0.000 | 0.000 |
| NAM | 0.000 | 0.000 | 0.000 | 0.000 | 0.000 | 0.000 |
| NER | 0.000 | 0.000 | 0.000 | 0.000 | 0.000 | 0.000 |
| NGA | 0.233 | 0.798 | 0.000 | 0.000 | 0.444 | 0.307 |
| RWA | 0.000 | 0.000 | 0.000 | 0.000 | 0.000 | 0.000 |
| SEN | 0.000 | 0.000 | 0.000 | 0.000 | 0.000 | 0.317 |
| SYC | 0.000 | 0.000 | 0.000 | 0.000 | 0.000 | 0.000 |
| SLE | 0.000 | 0.000 | 0.000 | 0.000 | 0.000 | 0.000 |
| ZAF | 1.000 | 1.000 | 0.176 | 0.000 | 0.000 | 1.000 |
| SSD | 0.000 | 0.000 | 0.000 | 0.000 | 0.000 | 0.000 |
| SDN | 0.000 | 0.000 | 0.000 | 0.000 | 0.000 | 0.000 |
| TZA | 0.000 | 0.015 | 0.000 | 0.000 | 0.000 | 0.000 |
| TGO | 0.000 | 0.000 | 0.000 | 0.000 | 0.000 | 0.000 |
| TUN | 0.000 | 0.000 | 0.000 | 0.000 | 0.000 | 0.000 |
| UGA | 0.000 | 0.000 | 0.000 | 0.000 | 0.108 | 0.000 |
| ZMB | 0.447 | 0.000 | 0.000 | 0.000 | 0.210 | 0.404 |
| ZWE | 0.089 | 0.000 | 0.000 | 0.000 | 0.000 | 0.000 |
| Standard deviation | 0.182 | 0.209 | 0.145 | 0.146 | 0.191 | 0.251 |

Table A10. The overall utility matrix for 2019 data

| **Country** | **C1** | **C2** | **C3** | **C4** | **C5** | **C6** |
| --- | --- | --- | --- | --- | --- | --- |
| DZA | 0.114 | 0.288 | 0.000 | 1.000 | 1.000 | 0.000 |
| AGO | 0.358 | 0.000 | 1.000 | 0.226 | 0.335 | 0.000 |
| BEN | 0.000 | 0.000 | 0.000 | 0.000 | 0.000 | 0.000 |
| BWA | 0.000 | 0.000 | 0.000 | 0.000 | 0.000 | 0.000 |
| BFA | 0.000 | 0.000 | 0.000 | 0.000 | 0.000 | 0.000 |
| BDI | 0.000 | 0.000 | 0.000 | 0.000 | 0.000 | 0.000 |
| CMR | 0.000 | 0.000 | 0.000 | 0.000 | 0.017 | 0.000 |
| CPV | 0.000 | 0.000 | 0.000 | 0.000 | 0.000 | 0.000 |
| CAF | 0.000 | 0.000 | 0.000 | 0.000 | 0.000 | 0.000 |
| TCD | 0.000 | 0.000 | 0.000 | 0.000 | 0.000 | 0.000 |
| COM | 0.000 | 0.000 | 0.000 | 0.000 | 0.000 | 0.000 |
| COG | 0.000 | 0.000 | 0.066 | 0.000 | 0.000 | 0.000 |
| COD | 0.897 | 0.000 | 0.000 | 0.000 | 0.247 | 0.000 |
| CIV | 0.000 | 0.000 | 0.000 | 0.000 | 0.000 | 0.083 |
| DJI | 0.000 | 0.000 | 0.000 | 0.000 | 0.000 | 0.000 |
| EGY | 0.000 | 0.688 | 0.000 | 0.000 | 0.401 | 1.000 |
| GNQ | 0.000 | 0.000 | 0.000 | 0.000 | 0.000 | 0.000 |
| ERI | 0.000 | 0.000 | 0.000 | 0.000 | 0.000 | 0.000 |
| ETH | 0.287 | 0.000 | 0.000 | 0.051 | 0.257 | 0.000 |
| GAB | 0.000 | 0.000 | 0.000 | 0.000 | 0.000 | 0.000 |
| GMB | 0.000 | 0.000 | 0.000 | 0.000 | 0.000 | 0.000 |
| GHA | 0.127 | 0.122 | 0.000 | 0.000 | 0.000 | 0.000 |
| GIN | 0.000 | 0.000 | 0.000 | 0.000 | 0.000 | 0.000 |
| KEN | 0.080 | 0.128 | 0.000 | 0.077 | 0.595 | 0.000 |
| LSO | 0.000 | 0.000 | 0.000 | 0.000 | 0.000 | 0.000 |
| LBR | 0.000 | 0.037 | 0.000 | 0.000 | 0.000 | 0.000 |
| MDG | 0.000 | 0.000 | 0.000 | 0.000 | 0.000 | 0.000 |
| MWI | 0.000 | 0.000 | 0.000 | 0.000 | 0.000 | 0.000 |
| MLI | 0.000 | 0.000 | 0.000 | 0.000 | 0.000 | 0.000 |
| MRT | 0.000 | 0.000 | 0.000 | 0.000 | 0.000 | 0.000 |
| MUS | 0.002 | 0.000 | 0.000 | 0.000 | 0.000 | 0.000 |
| MAR | 0.000 | 0.047 | 0.000 | 0.000 | 0.000 | 0.000 |
| MOZ | 0.000 | 0.000 | 0.000 | 0.000 | 0.000 | 0.000 |
| NAM | 0.000 | 0.000 | 0.000 | 0.000 | 0.000 | 0.000 |
| NER | 0.000 | 0.000 | 0.000 | 0.000 | 0.000 | 0.000 |
| NGA | 0.208 | 1.000 | 0.000 | 0.083 | 0.677 | 0.000 |
| RWA | 0.000 | 0.000 | 0.000 | 0.000 | 0.000 | 0.000 |
| SEN | 0.000 | 0.000 | 0.000 | 0.000 | 0.000 | 0.000 |
| SYC | 0.000 | 0.000 | 0.000 | 0.000 | 0.000 | 0.000 |
| SLE | 0.000 | 0.000 | 0.000 | 0.000 | 0.000 | 0.000 |
| ZAF | 1.000 | 0.995 | 0.281 | 0.000 | 0.000 | 0.000 |
| SSD | 0.000 | 0.000 | 0.000 | 0.000 | 0.000 | 0.000 |
| SDN | 0.000 | 0.000 | 0.000 | 0.000 | 0.000 | 0.000 |
| TZA | 0.012 | 0.028 | 0.000 | 0.000 | 0.000 | 0.000 |
| TGO | 0.000 | 0.000 | 0.000 | 0.000 | 0.000 | 0.000 |
| TUN | 0.000 | 0.000 | 0.000 | 0.000 | 0.000 | 0.000 |
| UGA | 0.000 | 0.000 | 0.000 | 0.000 | 0.050 | 0.000 |
| ZMB | 0.352 | 0.000 | 0.000 | 0.004 | 0.274 | 0.000 |
| ZWE | 0.113 | 0.000 | 0.000 | 0.000 | 0.000 | 0.000 |
| Standard deviation | 0.203 | 0.221 | 0.148 | 0.146 | 0.203 | 0.143 |

Table A11. The overall utility matrix for 2020 data

| **Country** | **C1** | **C2** | **C3** | **C4** | **C5** | **C6** |
| --- | --- | --- | --- | --- | --- | --- |
| DZA | 0.135 | 0.175 | 0.000 | 1.000 | 1.000 | 0.000 |
| AGO | 0.392 | 0.000 | 1.000 | 0.254 | 0.162 | 0.000 |
| BEN | 0.000 | 0.000 | 0.000 | 0.000 | 0.000 | 0.000 |
| BWA | 0.000 | 0.000 | 0.000 | 0.000 | 0.000 | 0.000 |
| BFA | 0.000 | 0.000 | 0.000 | 0.000 | 0.000 | 0.000 |
| BDI | 0.000 | 0.000 | 0.000 | 0.000 | 0.000 | 0.000 |
| CMR | 0.000 | 0.000 | 0.000 | 0.000 | 0.000 | 0.000 |
| CPV | 0.000 | 0.000 | 0.000 | 0.000 | 0.000 | 0.000 |
| CAF | 0.000 | 0.000 | 0.000 | 0.000 | 0.000 | 0.000 |
| TCD | 0.000 | 0.000 | 0.000 | 0.000 | 0.000 | 0.000 |
| COM | 0.000 | 0.000 | 0.000 | 0.000 | 0.000 | 0.000 |
| COG | 0.000 | 0.000 | 0.033 | 0.000 | 0.000 | 0.000 |
| COD | 0.623 | 0.000 | 0.376 | 0.263 | 0.308 | 0.131 |
| CIV | 0.000 | 0.000 | 0.000 | 0.000 | 0.000 | 0.000 |
| DJI | 0.000 | 0.000 | 0.000 | 0.000 | 0.000 | 0.000 |
| EGY | 0.016 | 0.781 | 0.000 | 0.000 | 0.571 | 0.000 |
| GNQ | 0.000 | 0.000 | 0.000 | 0.000 | 0.000 | 0.000 |
| ERI | 0.000 | 0.000 | 0.000 | 0.000 | 0.000 | 0.000 |
| ETH | 0.463 | 0.000 | 0.000 | 0.494 | 0.553 | 0.000 |
| GAB | 0.000 | 0.000 | 0.019 | 0.000 | 0.000 | 0.000 |
| GMB | 0.000 | 0.000 | 0.000 | 0.000 | 0.000 | 0.000 |
| GHA | 0.119 | 0.268 | 0.000 | 0.000 | 0.045 | 1.000 |
| GIN | 0.000 | 0.000 | 0.000 | 0.226 | 0.238 | 0.000 |
| KEN | 0.263 | 0.160 | 0.000 | 0.131 | 0.561 | 0.000 |
| LSO | 0.000 | 0.000 | 0.000 | 0.000 | 0.000 | 0.000 |
| LBR | 0.000 | 0.000 | 0.000 | 0.000 | 0.000 | 0.000 |
| MDG | 0.000 | 0.000 | 0.000 | 0.000 | 0.000 | 0.000 |
| MWI | 0.000 | 0.000 | 0.000 | 0.000 | 0.000 | 0.000 |
| MLI | 0.000 | 0.000 | 0.000 | 0.000 | 0.000 | 0.000 |
| MRT | 0.000 | 0.000 | 0.000 | 0.000 | 0.000 | 0.000 |
| MUS | 0.000 | 0.000 | 0.000 | 0.000 | 0.000 | 0.000 |
| MAR | 0.000 | 0.056 | 0.000 | 0.000 | 0.000 | 0.000 |
| MOZ | 0.050 | 0.000 | 0.000 | 0.000 | 0.000 | 0.080 |
| NAM | 0.000 | 0.000 | 0.000 | 0.000 | 0.000 | 0.000 |
| NER | 0.012 | 0.000 | 0.000 | 0.000 | 0.000 | 0.000 |
| NGA | 0.315 | 1.000 | 0.000 | 0.375 | 0.705 | 0.000 |
| RWA | 0.000 | 0.000 | 0.000 | 0.000 | 0.000 | 0.304 |
| SEN | 0.000 | 0.000 | 0.000 | 0.000 | 0.000 | 0.000 |
| SYC | 0.000 | 0.000 | 0.000 | 0.000 | 0.000 | 0.000 |
| SLE | 0.000 | 0.000 | 0.000 | 0.000 | 0.000 | 0.000 |
| ZAF | 1.000 | 0.894 | 0.616 | 0.000 | 0.000 | 0.000 |
| SSD | 0.000 | 0.000 | 0.000 | 0.000 | 0.000 | 0.000 |
| SDN | 0.000 | 0.000 | 0.000 | 0.000 | 0.000 | 0.000 |
| TZA | 0.108 | 0.056 | 0.000 | 0.017 | 0.145 | 0.000 |
| TGO | 0.000 | 0.000 | 0.000 | 0.000 | 0.000 | 0.000 |
| TUN | 0.000 | 0.000 | 0.000 | 0.000 | 0.000 | 0.000 |
| UGA | 0.000 | 0.000 | 0.000 | 0.000 | 0.074 | 0.519 |
| ZMB | 0.478 | 0.000 | 0.028 | 0.158 | 0.307 | 0.000 |
| ZWE | 0.173 | 0.000 | 0.000 | 0.000 | 0.000 | 0.000 |
| Standard deviation | 0.198 | 0.219 | 0.173 | 0.173 | 0.219 | 0.165 |

Table A12. The overall utility matrix for 2021 data

| **Country** | **C1** | **C2** | **C3** | **C4** | **C5** | **C6** |
| --- | --- | --- | --- | --- | --- | --- |
| DZA | 0.163 | 0.101 | 0.000 | 1.000 | 0.919 | 0.000 |
| AGO | 0.412 | 0.000 | 1.000 | 0.470 | 0.409 | 0.000 |
| BEN | 0.000 | 0.000 | 0.000 | 0.000 | 0.000 | 1.000 |
| BWA | 0.000 | 0.000 | 0.000 | 0.000 | 0.000 | 0.000 |
| BFA | 0.000 | 0.000 | 0.000 | 0.000 | 0.000 | 0.000 |
| BDI | 0.000 | 0.000 | 0.000 | 0.000 | 0.000 | 0.000 |
| CMR | 0.000 | 0.000 | 0.000 | 0.070 | 0.000 | 0.000 |
| CPV | 0.000 | 0.000 | 0.000 | 0.000 | 0.000 | 0.000 |
| CAF | 0.000 | 0.000 | 0.000 | 0.000 | 0.000 | 0.000 |
| TCD | 0.000 | 0.000 | 0.000 | 0.000 | 0.000 | 0.000 |
| COM | 0.000 | 0.000 | 0.000 | 0.000 | 0.000 | 0.000 |
| COG | 0.000 | 0.000 | 0.022 | 0.000 | 0.000 | 0.000 |
| COD | 0.772 | 0.000 | 0.471 | 0.624 | 0.400 | 0.000 |
| CIV | 0.000 | 0.000 | 0.000 | 0.026 | 0.072 | 0.000 |
| DJI | 0.000 | 0.000 | 0.000 | 0.000 | 0.000 | 0.000 |
| EGY | 0.046 | 0.774 | 0.000 | 0.465 | 0.361 | 0.000 |
| GNQ | 0.000 | 0.000 | 0.000 | 0.000 | 0.000 | 0.000 |
| ERI | 0.000 | 0.000 | 0.000 | 0.000 | 0.000 | 0.000 |
| ETH | 0.436 | 0.000 | 0.000 | 0.601 | 0.384 | 0.000 |
| GAB | 0.000 | 0.000 | 0.000 | 0.000 | 0.000 | 0.000 |
| GMB | 0.000 | 0.000 | 0.000 | 0.000 | 0.000 | 0.000 |
| GHA | 0.000 | 0.208 | 0.000 | 0.000 | 0.245 | 0.000 |
| GIN | 0.000 | 0.000 | 0.000 | 0.507 | 0.084 | 0.000 |
| KEN | 0.301 | 0.125 | 0.000 | 0.333 | 0.509 | 0.000 |
| LSO | 0.000 | 0.000 | 0.000 | 0.000 | 0.000 | 0.000 |
| LBR | 0.000 | 0.059 | 0.000 | 0.000 | 0.000 | 0.000 |
| MDG | 0.000 | 0.000 | 0.000 | 0.000 | 0.000 | 0.000 |
| MWI | 0.000 | 0.000 | 0.000 | 0.000 | 0.000 | 0.000 |
| MLI | 0.000 | 0.000 | 0.000 | 0.000 | 0.000 | 0.000 |
| MRT | 0.000 | 0.000 | 0.000 | 0.000 | 0.000 | 0.000 |
| MUS | 0.000 | 0.000 | 0.000 | 0.000 | 0.000 | 0.000 |
| MAR | 0.000 | 0.060 | 0.000 | 0.000 | 0.000 | 0.000 |
| MOZ | 0.043 | 0.000 | 0.000 | 0.000 | 0.000 | 0.000 |
| NAM | 0.000 | 0.000 | 0.000 | 0.000 | 0.000 | 0.000 |
| NER | 0.086 | 0.000 | 0.000 | 0.000 | 0.000 | 0.000 |
| NGA | 0.408 | 1.000 | 0.000 | 0.656 | 1.000 | 0.000 |
| RWA | 0.000 | 0.000 | 0.000 | 0.000 | 0.000 | 0.000 |
| SEN | 0.000 | 0.000 | 0.000 | 0.000 | 0.000 | 0.596 |
| SYC | 0.000 | 0.000 | 0.000 | 0.000 | 0.000 | 0.000 |
| SLE | 0.000 | 0.000 | 0.000 | 0.000 | 0.000 | 0.000 |
| ZAF | 1.000 | 0.923 | 0.584 | 0.000 | 0.000 | 0.000 |
| SSD | 0.000 | 0.000 | 0.000 | 0.000 | 0.000 | 0.000 |
| SDN | 0.003 | 0.000 | 0.000 | 0.000 | 0.000 | 0.000 |
| TZA | 0.127 | 0.088 | 0.000 | 0.227 | 0.272 | 0.000 |
| TGO | 0.000 | 0.000 | 0.000 | 0.000 | 0.000 | 0.000 |
| TUN | 0.000 | 0.000 | 0.000 | 0.000 | 0.000 | 0.000 |
| UGA | 0.000 | 0.000 | 0.000 | 0.032 | 0.068 | 0.623 |
| ZMB | 0.488 | 0.000 | 0.002 | 0.290 | 0.135 | 0.000 |
| ZWE | 0.161 | 0.000 | 0.000 | 0.000 | 0.000 | 0.000 |
| Standard deviation | 0.211 | 0.219 | 0.175 | 0.231 | 0.223 | 0.185 |

Table A13. The theoretical evaluation matrix for 2018 data

| **Country** | **C1** | **C2** | **C3** | **C4** | **C5** | **C6** |
| --- | --- | --- | --- | --- | --- | --- |
| DZA | 0.005 | 0.004 | 0.001 | 0.000 | 0.005 | 0.0060 |
| AGO | 0.005 | 0.004 | 0.001 | 0.000 | 0.005 | 0.0060 |
| BEN | 0.005 | 0.004 | 0.001 | 0.000 | 0.005 | 0.0060 |
| BWA | 0.005 | 0.004 | 0.001 | 0.000 | 0.005 | 0.0060 |
| BFA | 0.005 | 0.004 | 0.001 | 0.000 | 0.005 | 0.0060 |
| BDI | 0.005 | 0.004 | 0.001 | 0.000 | 0.005 | 0.0060 |
| CMR | 0.005 | 0.004 | 0.001 | 0.000 | 0.005 | 0.0060 |
| CPV | 0.005 | 0.004 | 0.001 | 0.000 | 0.005 | 0.0060 |
| CAF | 0.005 | 0.004 | 0.001 | 0.000 | 0.005 | 0.0060 |
| TCD | 0.005 | 0.004 | 0.001 | 0.000 | 0.005 | 0.0060 |
| COM | 0.005 | 0.004 | 0.001 | 0.000 | 0.005 | 0.0060 |
| COG | 0.005 | 0.004 | 0.001 | 0.000 | 0.005 | 0.0060 |
| COD | 0.005 | 0.004 | 0.001 | 0.000 | 0.005 | 0.0060 |
| CIV | 0.005 | 0.004 | 0.001 | 0.000 | 0.005 | 0.0060 |
| DJI | 0.005 | 0.004 | 0.001 | 0.000 | 0.005 | 0.0060 |
| EGY | 0.005 | 0.004 | 0.001 | 0.000 | 0.005 | 0.0060 |
| GNQ | 0.005 | 0.004 | 0.001 | 0.000 | 0.005 | 0.0060 |
| ERI | 0.005 | 0.004 | 0.001 | 0.000 | 0.005 | 0.0060 |
| ETH | 0.005 | 0.004 | 0.001 | 0.000 | 0.005 | 0.0060 |
| GAB | 0.005 | 0.004 | 0.001 | 0.000 | 0.005 | 0.0060 |
| GMB | 0.005 | 0.004 | 0.001 | 0.000 | 0.005 | 0.0060 |
| GHA | 0.005 | 0.004 | 0.001 | 0.000 | 0.005 | 0.0060 |
| GIN | 0.005 | 0.004 | 0.001 | 0.000 | 0.005 | 0.0060 |
| KEN | 0.005 | 0.004 | 0.001 | 0.000 | 0.005 | 0.0060 |
| LSO | 0.005 | 0.004 | 0.001 | 0.000 | 0.005 | 0.0060 |
| LBR | 0.005 | 0.004 | 0.001 | 0.000 | 0.005 | 0.0060 |
| MDG | 0.005 | 0.004 | 0.001 | 0.000 | 0.005 | 0.0060 |
| MWI | 0.005 | 0.004 | 0.001 | 0.000 | 0.005 | 0.0060 |
| MLI | 0.005 | 0.004 | 0.001 | 0.000 | 0.005 | 0.0060 |
| MRT | 0.005 | 0.004 | 0.001 | 0.000 | 0.005 | 0.0060 |
| MUS | 0.005 | 0.004 | 0.001 | 0.000 | 0.005 | 0.0060 |
| MAR | 0.005 | 0.004 | 0.001 | 0.000 | 0.005 | 0.0060 |
| MOZ | 0.005 | 0.004 | 0.001 | 0.000 | 0.005 | 0.0060 |
| NAM | 0.005 | 0.004 | 0.001 | 0.000 | 0.005 | 0.0060 |
| NER | 0.005 | 0.004 | 0.001 | 0.000 | 0.005 | 0.0060 |
| NGA | 0.005 | 0.004 | 0.001 | 0.000 | 0.005 | 0.0060 |
| RWA | 0.005 | 0.004 | 0.001 | 0.000 | 0.005 | 0.0060 |
| SEN | 0.005 | 0.004 | 0.001 | 0.000 | 0.005 | 0.0060 |
| SYC | 0.005 | 0.004 | 0.001 | 0.000 | 0.005 | 0.0060 |
| SLE | 0.005 | 0.004 | 0.001 | 0.000 | 0.005 | 0.0060 |
| ZAF | 0.005 | 0.004 | 0.001 | 0.000 | 0.005 | 0.0060 |
| SSD | 0.005 | 0.004 | 0.001 | 0.000 | 0.005 | 0.0060 |
| SDN | 0.005 | 0.004 | 0.001 | 0.000 | 0.005 | 0.0060 |
| TZA | 0.005 | 0.004 | 0.001 | 0.000 | 0.005 | 0.0060 |
| TGO | 0.005 | 0.004 | 0.001 | 0.000 | 0.005 | 0.0060 |
| TUN | 0.005 | 0.004 | 0.001 | 0.000 | 0.005 | 0.0060 |
| UGA | 0.005 | 0.004 | 0.001 | 0.000 | 0.005 | 0.0060 |
| ZMB | 0.005 | 0.004 | 0.001 | 0.000 | 0.005 | 0.0060 |
| ZWE | 0.005 | 0.004 | 0.001 | 0.000 | 0.005 | 0.0060 |

Table A14. The observational evaluation matrix for 2018 data

| **Country** | **C1** | **C2** | **C3** | **C4** | **C5** | **C6** |
| --- | --- | --- | --- | --- | --- | --- |
| DZA | 0.0016 | 0.0017 | 0.0000 | 0.0005 | 0.0049 | 0.0000 |
| AGO | 0.0017 | 0.0005 | 0.0006 | 0.0002 | 0.0030 | 0.0038 |
| BEN | 0.0001 | 0.0005 | 0.0000 | 0.0000 | 0.0001 | 0.0000 |
| BWA | 0.0002 | 0.0001 | 0.0000 | 0.0000 | 0.0002 | 0.0000 |
| BFA | 0.0000 | 0.0000 | 0.0000 | 0.0000 | 0.0000 | 0.0000 |
| BDI | 0.0000 | 0.0000 | 0.0000 | 0.0000 | 0.0000 | 0.0000 |
| CMR | 0.0004 | 0.0004 | 0.0000 | 0.0000 | 0.0008 | 0.0005 |
| CPV | 0.0000 | 0.0000 | 0.0000 | 0.0000 | 0.0000 | 0.0000 |
| CAF | 0.0001 | 0.0000 | 0.0000 | 0.0000 | 0.0000 | 0.0000 |
| TCD | 0.0004 | 0.0000 | 0.0000 | 0.0000 | 0.0003 | 0.0000 |
| COM | 0.0000 | 0.0000 | 0.0000 | 0.0000 | 0.0000 | 0.0002 |
| COG | 0.0006 | 0.0001 | 0.0001 | 0.0000 | 0.0003 | 0.0000 |
| COD | 0.0033 | 0.0004 | 0.0001 | 0.0001 | 0.0012 | 0.0020 |
| CIV | 0.0003 | 0.0004 | 0.0000 | 0.0000 | 0.0006 | 0.0008 |
| DJI | 0.0001 | 0.0004 | 0.0000 | 0.0000 | 0.0002 | 0.0000 |
| EGY | 0.0008 | 0.0026 | 0.0000 | 0.0000 | 0.0013 | 0.0054 |
| GNQ | 0.0004 | 0.0000 | 0.0000 | 0.0000 | 0.0003 | 0.0000 |
| ERI | 0.0002 | 0.0000 | 0.0000 | 0.0000 | 0.0000 | 0.0003 |
| ETH | 0.0019 | 0.0005 | 0.0000 | 0.0001 | 0.0026 | 0.0007 |
| GAB | 0.0002 | 0.0001 | 0.0001 | 0.0000 | 0.0001 | 0.0002 |
| GMB | 0.0000 | 0.0001 | 0.0000 | 0.0000 | 0.0000 | 0.0001 |
| GHA | 0.0014 | 0.0010 | 0.0001 | 0.0000 | 0.0007 | 0.0019 |
| GIN | 0.0006 | 0.0003 | 0.0000 | 0.0000 | 0.0005 | 0.0059 |
| KEN | 0.0013 | 0.0011 | 0.0000 | 0.0001 | 0.0029 | 0.0005 |
| LSO | 0.0000 | 0.0000 | 0.0000 | 0.0000 | 0.0000 | 0.0003 |
| LBR | 0.0002 | 0.0004 | 0.0000 | 0.0000 | 0.0001 | 0.0000 |
| MDG | 0.0006 | 0.0002 | 0.0000 | 0.0000 | 0.0001 | 0.0003 |
| MWI | 0.0002 | 0.0000 | 0.0000 | 0.0000 | 0.0001 | 0.0000 |
| MLI | 0.0002 | 0.0001 | 0.0000 | 0.0000 | 0.0003 | 0.0005 |
| MRT | 0.0002 | 0.0002 | 0.0000 | 0.0000 | 0.0001 | 0.0000 |
| MUS | 0.0008 | 0.0002 | 0.0000 | 0.0000 | 0.0001 | 0.0000 |
| MAR | 0.0003 | 0.0008 | 0.0000 | 0.0000 | 0.0004 | 0.0004 |
| MOZ | 0.0011 | 0.0004 | 0.0000 | 0.0000 | 0.0005 | 0.0000 |
| NAM | 0.0003 | 0.0001 | 0.0000 | 0.0000 | 0.0003 | 0.0000 |
| NER | 0.0006 | 0.0000 | 0.0000 | 0.0000 | 0.0001 | 0.0000 |
| NGA | 0.0018 | 0.0029 | 0.0000 | 0.0001 | 0.0027 | 0.0026 |
| RWA | 0.0001 | 0.0000 | 0.0000 | 0.0000 | 0.0002 | 0.0004 |
| SEN | 0.0002 | 0.0005 | 0.0000 | 0.0000 | 0.0007 | 0.0026 |
| SYC | 0.0003 | 0.0000 | 0.0000 | 0.0000 | 0.0000 | 0.0000 |
| SLE | 0.0001 | 0.0001 | 0.0000 | 0.0000 | 0.0001 | 0.0000 |
| ZAF | 0.0049 | 0.0035 | 0.0002 | 0.0000 | 0.0009 | 0.0060 |
| SSD | 0.0000 | 0.0000 | 0.0000 | 0.0000 | 0.0003 | 0.0007 |
| SDN | 0.0010 | 0.0004 | 0.0000 | 0.0000 | 0.0003 | 0.0001 |
| TZA | 0.0010 | 0.0008 | 0.0000 | 0.0000 | 0.0007 | 0.0000 |
| TGO | 0.0001 | 0.0004 | 0.0000 | 0.0000 | 0.0001 | 0.0000 |
| TUN | 0.0000 | 0.0003 | 0.0000 | 0.0000 | 0.0001 | 0.0000 |
| UGA | 0.0006 | 0.0001 | 0.0000 | 0.0000 | 0.0014 | 0.0000 |
| ZMB | 0.0027 | 0.0002 | 0.0001 | 0.0001 | 0.0018 | 0.0030 |
| ZWE | 0.0013 | 0.0001 | 0.0000 | 0.0000 | 0.0002 | 0.0005 |

Table A15. The theoretical evaluation matrix for 2019 data

| **Country** | **C1** | **C2** | **C3** | **C4** | **C5** | **C6** |
| --- | --- | --- | --- | --- | --- | --- |
| DZA | 0.006 | 0.004 | 0.001 | 0.001 | 0.007 | 0.0002 |
| AGO | 0.006 | 0.004 | 0.001 | 0.001 | 0.007 | 0.0002 |
| BEN | 0.006 | 0.004 | 0.001 | 0.001 | 0.007 | 0.0002 |
| BWA | 0.006 | 0.004 | 0.001 | 0.001 | 0.007 | 0.0002 |
| BFA | 0.006 | 0.004 | 0.001 | 0.001 | 0.007 | 0.0002 |
| BDI | 0.006 | 0.004 | 0.001 | 0.001 | 0.007 | 0.0002 |
| CMR | 0.006 | 0.004 | 0.001 | 0.001 | 0.007 | 0.0002 |
| CPV | 0.006 | 0.004 | 0.001 | 0.001 | 0.007 | 0.0002 |
| CAF | 0.006 | 0.004 | 0.001 | 0.001 | 0.007 | 0.0002 |
| TCD | 0.006 | 0.004 | 0.001 | 0.001 | 0.007 | 0.0002 |
| COM | 0.006 | 0.004 | 0.001 | 0.001 | 0.007 | 0.0002 |
| COG | 0.006 | 0.004 | 0.001 | 0.001 | 0.007 | 0.0002 |
| COD | 0.006 | 0.004 | 0.001 | 0.001 | 0.007 | 0.0002 |
| CIV | 0.006 | 0.004 | 0.001 | 0.001 | 0.007 | 0.0002 |
| DJI | 0.006 | 0.004 | 0.001 | 0.001 | 0.007 | 0.0002 |
| EGY | 0.006 | 0.004 | 0.001 | 0.001 | 0.007 | 0.0002 |
| GNQ | 0.006 | 0.004 | 0.001 | 0.001 | 0.007 | 0.0002 |
| ERI | 0.006 | 0.004 | 0.001 | 0.001 | 0.007 | 0.0002 |
| ETH | 0.006 | 0.004 | 0.001 | 0.001 | 0.007 | 0.0002 |
| GAB | 0.006 | 0.004 | 0.001 | 0.001 | 0.007 | 0.0002 |
| GMB | 0.006 | 0.004 | 0.001 | 0.001 | 0.007 | 0.0002 |
| GHA | 0.006 | 0.004 | 0.001 | 0.001 | 0.007 | 0.0002 |
| GIN | 0.006 | 0.004 | 0.001 | 0.001 | 0.007 | 0.0002 |
| KEN | 0.006 | 0.004 | 0.001 | 0.001 | 0.007 | 0.0002 |
| LSO | 0.006 | 0.004 | 0.001 | 0.001 | 0.007 | 0.0002 |
| LBR | 0.006 | 0.004 | 0.001 | 0.001 | 0.007 | 0.0002 |
| MDG | 0.006 | 0.004 | 0.001 | 0.001 | 0.007 | 0.0002 |
| MWI | 0.006 | 0.004 | 0.001 | 0.001 | 0.007 | 0.0002 |
| MLI | 0.006 | 0.004 | 0.001 | 0.001 | 0.007 | 0.0002 |
| MRT | 0.006 | 0.004 | 0.001 | 0.001 | 0.007 | 0.0002 |
| MUS | 0.006 | 0.004 | 0.001 | 0.001 | 0.007 | 0.0002 |
| MAR | 0.006 | 0.004 | 0.001 | 0.001 | 0.007 | 0.0002 |
| MOZ | 0.006 | 0.004 | 0.001 | 0.001 | 0.007 | 0.0002 |
| NAM | 0.006 | 0.004 | 0.001 | 0.001 | 0.007 | 0.0002 |
| NER | 0.006 | 0.004 | 0.001 | 0.001 | 0.007 | 0.0002 |
| NGA | 0.006 | 0.004 | 0.001 | 0.001 | 0.007 | 0.0002 |
| RWA | 0.006 | 0.004 | 0.001 | 0.001 | 0.007 | 0.0002 |
| SEN | 0.006 | 0.004 | 0.001 | 0.001 | 0.007 | 0.0002 |
| SYC | 0.006 | 0.004 | 0.001 | 0.001 | 0.007 | 0.0002 |
| SLE | 0.006 | 0.004 | 0.001 | 0.001 | 0.007 | 0.0002 |
| ZAF | 0.006 | 0.004 | 0.001 | 0.001 | 0.007 | 0.0002 |
| SSD | 0.006 | 0.004 | 0.001 | 0.001 | 0.007 | 0.0002 |
| SDN | 0.006 | 0.004 | 0.001 | 0.001 | 0.007 | 0.0002 |
| TZA | 0.006 | 0.004 | 0.001 | 0.001 | 0.007 | 0.0002 |
| TGO | 0.006 | 0.004 | 0.001 | 0.001 | 0.007 | 0.0002 |
| TUN | 0.006 | 0.004 | 0.001 | 0.001 | 0.007 | 0.0002 |
| UGA | 0.006 | 0.004 | 0.001 | 0.001 | 0.007 | 0.0002 |
| ZMB | 0.006 | 0.004 | 0.001 | 0.001 | 0.007 | 0.0002 |
| ZWE | 0.006 | 0.004 | 0.001 | 0.001 | 0.007 | 0.0002 |

Table A16. The observational evaluation matrix for 2019 data

| **Country** | **C1** | **C2** | **C3** | **C4** | **C5** | **C6** |
| --- | --- | --- | --- | --- | --- | --- |
| DZA | 0.0018 | 0.0018 | 0.0000 | 0.0012 | 0.0075 | 0.0000 |
| AGO | 0.0029 | 0.0005 | 0.0009 | 0.0005 | 0.0034 | 0.0000 |
| BEN | 0.0001 | 0.0006 | 0.0000 | 0.0000 | 0.0003 | 0.0000 |
| BWA | 0.0002 | 0.0001 | 0.0000 | 0.0000 | 0.0002 | 0.0000 |
| BFA | 0.0000 | 0.0001 | 0.0000 | 0.0000 | 0.0000 | 0.0000 |
| BDI | 0.0000 | 0.0000 | 0.0000 | 0.0000 | 0.0001 | 0.0000 |
| CMR | 0.0003 | 0.0004 | 0.0000 | 0.0001 | 0.0016 | 0.0000 |
| CPV | 0.0000 | 0.0000 | 0.0000 | 0.0000 | 0.0001 | 0.0000 |
| CAF | 0.0000 | 0.0000 | 0.0000 | 0.0000 | 0.0001 | 0.0000 |
| TCD | 0.0007 | 0.0001 | 0.0000 | 0.0001 | 0.0009 | 0.0000 |
| COM | 0.0000 | 0.0000 | 0.0000 | 0.0000 | 0.0000 | 0.0000 |
| COG | 0.0006 | 0.0001 | 0.0002 | 0.0001 | 0.0004 | 0.0000 |
| COD | 0.0057 | 0.0005 | 0.0002 | 0.0002 | 0.0029 | 0.0000 |
| CIV | 0.0006 | 0.0005 | 0.0000 | 0.0001 | 0.0013 | 0.0001 |
| DJI | 0.0001 | 0.0006 | 0.0000 | 0.0000 | 0.0003 | 0.0000 |
| EGY | 0.0011 | 0.0032 | 0.0000 | 0.0001 | 0.0038 | 0.0002 |
| GNQ | 0.0004 | 0.0000 | 0.0001 | 0.0001 | 0.0007 | 0.0000 |
| ERI | 0.0002 | 0.0000 | 0.0000 | 0.0000 | 0.0000 | 0.0000 |
| ETH | 0.0026 | 0.0006 | 0.0000 | 0.0003 | 0.0029 | 0.0000 |
| GAB | 0.0003 | 0.0001 | 0.0002 | 0.0000 | 0.0003 | 0.0000 |
| GMB | 0.0000 | 0.0001 | 0.0000 | 0.0000 | 0.0001 | 0.0000 |
| GHA | 0.0019 | 0.0013 | 0.0001 | 0.0001 | 0.0010 | 0.0000 |
| GIN | 0.0008 | 0.0004 | 0.0001 | 0.0002 | 0.0014 | 0.0000 |
| KEN | 0.0017 | 0.0013 | 0.0000 | 0.0003 | 0.0049 | 0.0000 |
| LSO | 0.0000 | 0.0000 | 0.0000 | 0.0000 | 0.0001 | 0.0000 |
| LBR | 0.0002 | 0.0010 | 0.0000 | 0.0000 | 0.0005 | 0.0000 |
| MDG | 0.0003 | 0.0003 | 0.0000 | 0.0000 | 0.0001 | 0.0000 |
| MWI | 0.0002 | 0.0001 | 0.0000 | 0.0000 | 0.0002 | 0.0000 |
| MLI | 0.0003 | 0.0001 | 0.0000 | 0.0000 | 0.0005 | 0.0000 |
| MRT | 0.0002 | 0.0003 | 0.0000 | 0.0000 | 0.0003 | 0.0000 |
| MUS | 0.0013 | 0.0002 | 0.0000 | 0.0000 | 0.0001 | 0.0000 |
| MAR | 0.0003 | 0.0011 | 0.0000 | 0.0000 | 0.0004 | 0.0000 |
| MOZ | 0.0012 | 0.0005 | 0.0000 | 0.0001 | 0.0012 | 0.0000 |
| NAM | 0.0004 | 0.0000 | 0.0000 | 0.0000 | 0.0005 | 0.0000 |
| NER | 0.0010 | 0.0001 | 0.0000 | 0.0000 | 0.0003 | 0.0000 |
| NGA | 0.0022 | 0.0044 | 0.0001 | 0.0003 | 0.0054 | 0.0000 |
| RWA | 0.0002 | 0.0001 | 0.0000 | 0.0000 | 0.0004 | 0.0000 |
| SEN | 0.0002 | 0.0006 | 0.0000 | 0.0001 | 0.0010 | 0.0000 |
| SYC | 0.0004 | 0.0000 | 0.0000 | 0.0000 | 0.0000 | 0.0000 |
| SLE | 0.0002 | 0.0001 | 0.0000 | 0.0000 | 0.0001 | 0.0000 |
| ZAF | 0.0063 | 0.0044 | 0.0004 | 0.0000 | 0.0007 | 0.0000 |
| SSD | 0.0000 | 0.0000 | 0.0001 | 0.0000 | 0.0003 | 0.0000 |
| SDN | 0.0012 | 0.0006 | 0.0000 | 0.0001 | 0.0004 | 0.0000 |
| TZA | 0.0014 | 0.0010 | 0.0000 | 0.0002 | 0.0015 | 0.0000 |
| TGO | 0.0001 | 0.0006 | 0.0000 | 0.0000 | 0.0001 | 0.0000 |
| TUN | 0.0000 | 0.0004 | 0.0000 | 0.0000 | 0.0001 | 0.0000 |
| UGA | 0.0007 | 0.0002 | 0.0000 | 0.0002 | 0.0018 | 0.0000 |
| ZMB | 0.0029 | 0.0002 | 0.0001 | 0.0003 | 0.0030 | 0.0000 |
| ZWE | 0.0018 | 0.0001 | 0.0000 | 0.0000 | 0.0007 | 0.0000 |

Table A17. The theoretical evaluation matrix for 2020 data

| **Country** | **C1** | **C2** | **C3** | **C4** | **C5** | **C6** |
| --- | --- | --- | --- | --- | --- | --- |
| DZA | 0.006 | 0.003 | 0.001 | 0.003 | 0.006 | 0.0015 |
| AGO | 0.006 | 0.003 | 0.001 | 0.003 | 0.006 | 0.0015 |
| BEN | 0.006 | 0.003 | 0.001 | 0.003 | 0.006 | 0.0015 |
| BWA | 0.006 | 0.003 | 0.001 | 0.003 | 0.006 | 0.0015 |
| BFA | 0.006 | 0.003 | 0.001 | 0.003 | 0.006 | 0.0015 |
| BDI | 0.006 | 0.003 | 0.001 | 0.003 | 0.006 | 0.0015 |
| CMR | 0.006 | 0.003 | 0.001 | 0.003 | 0.006 | 0.0015 |
| CPV | 0.006 | 0.003 | 0.001 | 0.003 | 0.006 | 0.0015 |
| CAF | 0.006 | 0.003 | 0.001 | 0.003 | 0.006 | 0.0015 |
| TCD | 0.006 | 0.003 | 0.001 | 0.003 | 0.006 | 0.0015 |
| COM | 0.006 | 0.003 | 0.001 | 0.003 | 0.006 | 0.0015 |
| COG | 0.006 | 0.003 | 0.001 | 0.003 | 0.006 | 0.0015 |
| COD | 0.006 | 0.003 | 0.001 | 0.003 | 0.006 | 0.0015 |
| CIV | 0.006 | 0.003 | 0.001 | 0.003 | 0.006 | 0.0015 |
| DJI | 0.006 | 0.003 | 0.001 | 0.003 | 0.006 | 0.0015 |
| EGY | 0.006 | 0.003 | 0.001 | 0.003 | 0.006 | 0.0015 |
| GNQ | 0.006 | 0.003 | 0.001 | 0.003 | 0.006 | 0.0015 |
| ERI | 0.006 | 0.003 | 0.001 | 0.003 | 0.006 | 0.0015 |
| ETH | 0.006 | 0.003 | 0.001 | 0.003 | 0.006 | 0.0015 |
| GAB | 0.006 | 0.003 | 0.001 | 0.003 | 0.006 | 0.0015 |
| GMB | 0.006 | 0.003 | 0.001 | 0.003 | 0.006 | 0.0015 |
| GHA | 0.006 | 0.003 | 0.001 | 0.003 | 0.006 | 0.0015 |
| GIN | 0.006 | 0.003 | 0.001 | 0.003 | 0.006 | 0.0015 |
| KEN | 0.006 | 0.003 | 0.001 | 0.003 | 0.006 | 0.0015 |
| LSO | 0.006 | 0.003 | 0.001 | 0.003 | 0.006 | 0.0015 |
| LBR | 0.006 | 0.003 | 0.001 | 0.003 | 0.006 | 0.0015 |
| MDG | 0.006 | 0.003 | 0.001 | 0.003 | 0.006 | 0.0015 |
| MWI | 0.006 | 0.003 | 0.001 | 0.003 | 0.006 | 0.0015 |
| MLI | 0.006 | 0.003 | 0.001 | 0.003 | 0.006 | 0.0015 |
| MRT | 0.006 | 0.003 | 0.001 | 0.003 | 0.006 | 0.0015 |
| MUS | 0.006 | 0.003 | 0.001 | 0.003 | 0.006 | 0.0015 |
| MAR | 0.006 | 0.003 | 0.001 | 0.003 | 0.006 | 0.0015 |
| MOZ | 0.006 | 0.003 | 0.001 | 0.003 | 0.006 | 0.0015 |
| NAM | 0.006 | 0.003 | 0.001 | 0.003 | 0.006 | 0.0015 |
| NER | 0.006 | 0.003 | 0.001 | 0.003 | 0.006 | 0.0015 |
| NGA | 0.006 | 0.003 | 0.001 | 0.003 | 0.006 | 0.0015 |
| RWA | 0.006 | 0.003 | 0.001 | 0.003 | 0.006 | 0.0015 |
| SEN | 0.006 | 0.003 | 0.001 | 0.003 | 0.006 | 0.0015 |
| SYC | 0.006 | 0.003 | 0.001 | 0.003 | 0.006 | 0.0015 |
| SLE | 0.006 | 0.003 | 0.001 | 0.003 | 0.006 | 0.0015 |
| ZAF | 0.006 | 0.003 | 0.001 | 0.003 | 0.006 | 0.0015 |
| SSD | 0.006 | 0.003 | 0.001 | 0.003 | 0.006 | 0.0015 |
| SDN | 0.006 | 0.003 | 0.001 | 0.003 | 0.006 | 0.0015 |
| TZA | 0.006 | 0.003 | 0.001 | 0.003 | 0.006 | 0.0015 |
| TGO | 0.006 | 0.003 | 0.001 | 0.003 | 0.006 | 0.0015 |
| TUN | 0.006 | 0.003 | 0.001 | 0.003 | 0.006 | 0.0015 |
| UGA | 0.006 | 0.003 | 0.001 | 0.003 | 0.006 | 0.0015 |
| ZMB | 0.006 | 0.003 | 0.001 | 0.003 | 0.006 | 0.0015 |
| ZWE | 0.006 | 0.003 | 0.001 | 0.003 | 0.006 | 0.0015 |

Table A18. The observational evaluation matrix for 2020 data

| **Country** | **C1** | **C2** | **C3** | **C4** | **C5** | **C6** |
| --- | --- | --- | --- | --- | --- | --- |
| DZA | 0.0017 | 0.0009 | 0.0001 | 0.0034 | 0.0057 | 0.0000 |
| AGO | 0.0027 | 0.0003 | 0.0014 | 0.0013 | 0.0018 | 0.0000 |
| BEN | 0.0001 | 0.0002 | 0.0000 | 0.0002 | 0.0003 | 0.0000 |
| BWA | 0.0002 | 0.0000 | 0.0000 | 0.0001 | 0.0002 | 0.0000 |
| BFA | 0.0000 | 0.0000 | 0.0000 | 0.0000 | 0.0000 | 0.0002 |
| BDI | 0.0000 | 0.0000 | 0.0000 | 0.0001 | 0.0001 | 0.0000 |
| CMR | 0.0004 | 0.0003 | 0.0001 | 0.0006 | 0.0011 | 0.0000 |
| CPV | 0.0000 | 0.0000 | 0.0000 | 0.0000 | 0.0000 | 0.0000 |
| CAF | 0.0000 | 0.0000 | 0.0000 | 0.0001 | 0.0001 | 0.0000 |
| TCD | 0.0007 | 0.0000 | 0.0000 | 0.0004 | 0.0007 | 0.0000 |
| COM | 0.0000 | 0.0000 | 0.0000 | 0.0000 | 0.0000 | 0.0000 |
| COG | 0.0012 | 0.0001 | 0.0003 | 0.0003 | 0.0007 | 0.0000 |
| COD | 0.0038 | 0.0003 | 0.0007 | 0.0014 | 0.0025 | 0.0005 |
| CIV | 0.0007 | 0.0004 | 0.0001 | 0.0003 | 0.0011 | 0.0002 |
| DJI | 0.0001 | 0.0004 | 0.0000 | 0.0001 | 0.0001 | 0.0000 |
| EGY | 0.0012 | 0.0023 | 0.0001 | 0.0004 | 0.0037 | 0.0000 |
| GNQ | 0.0003 | 0.0000 | 0.0001 | 0.0003 | 0.0004 | 0.0000 |
| ERI | 0.0002 | 0.0000 | 0.0000 | 0.0000 | 0.0000 | 0.0000 |
| ETH | 0.0030 | 0.0004 | 0.0000 | 0.0020 | 0.0036 | 0.0000 |
| GAB | 0.0003 | 0.0001 | 0.0003 | 0.0001 | 0.0001 | 0.0000 |
| GMB | 0.0000 | 0.0001 | 0.0000 | 0.0001 | 0.0001 | 0.0000 |
| GHA | 0.0016 | 0.0011 | 0.0002 | 0.0005 | 0.0014 | 0.0015 |
| GIN | 0.0005 | 0.0003 | 0.0002 | 0.0013 | 0.0022 | 0.0000 |
| KEN | 0.0022 | 0.0009 | 0.0000 | 0.0010 | 0.0036 | 0.0000 |
| LSO | 0.0000 | 0.0000 | 0.0000 | 0.0000 | 0.0001 | 0.0002 |
| LBR | 0.0002 | 0.0006 | 0.0000 | 0.0001 | 0.0003 | 0.0000 |
| MDG | 0.0004 | 0.0002 | 0.0000 | 0.0001 | 0.0002 | 0.0002 |
| MWI | 0.0002 | 0.0000 | 0.0000 | 0.0001 | 0.0002 | 0.0003 |
| MLI | 0.0003 | 0.0001 | 0.0000 | 0.0002 | 0.0003 | 0.0000 |
| MRT | 0.0002 | 0.0001 | 0.0001 | 0.0001 | 0.0002 | 0.0000 |
| MUS | 0.0009 | 0.0001 | 0.0000 | 0.0001 | 0.0002 | 0.0000 |
| MAR | 0.0004 | 0.0007 | 0.0001 | 0.0001 | 0.0003 | 0.0000 |
| MOZ | 0.0013 | 0.0003 | 0.0001 | 0.0006 | 0.0008 | 0.0004 |
| NAM | 0.0004 | 0.0000 | 0.0001 | 0.0001 | 0.0003 | 0.0000 |
| NER | 0.0012 | 0.0000 | 0.0000 | 0.0003 | 0.0005 | 0.0000 |
| NGA | 0.0024 | 0.0028 | 0.0002 | 0.0017 | 0.0043 | 0.0000 |
| RWA | 0.0002 | 0.0000 | 0.0000 | 0.0002 | 0.0004 | 0.0007 |
| SEN | 0.0004 | 0.0004 | 0.0000 | 0.0002 | 0.0006 | 0.0000 |
| SYC | 0.0004 | 0.0000 | 0.0000 | 0.0000 | 0.0000 | 0.0000 |
| SLE | 0.0001 | 0.0001 | 0.0000 | 0.0001 | 0.0001 | 0.0000 |
| ZAF | 0.0055 | 0.0025 | 0.0010 | 0.0000 | 0.0006 | 0.0000 |
| SSD | 0.0000 | 0.0000 | 0.0001 | 0.0002 | 0.0004 | 0.0000 |
| SDN | 0.0011 | 0.0004 | 0.0001 | 0.0003 | 0.0004 | 0.0000 |
| TZA | 0.0016 | 0.0007 | 0.0000 | 0.0008 | 0.0018 | 0.0000 |
| TGO | 0.0001 | 0.0004 | 0.0000 | 0.0000 | 0.0001 | 0.0000 |
| TUN | 0.0000 | 0.0002 | 0.0000 | 0.0000 | 0.0001 | 0.0000 |
| UGA | 0.0007 | 0.0001 | 0.0000 | 0.0005 | 0.0015 | 0.0009 |
| ZMB | 0.0031 | 0.0001 | 0.0003 | 0.0011 | 0.0025 | 0.0003 |
| ZWE | 0.0018 | 0.0001 | 0.0001 | 0.0003 | 0.0009 | 0.0000 |

Table A19. The theoretical evaluation matrix for 2021 data

| **Country** | **C1** | **C2** | **C3** | **C4** | **C5** | **C6** |
| --- | --- | --- | --- | --- | --- | --- |
| DZA | 0.005 | 0.002 | 0.001 | 0.006 | 0.005 | 0.0012 |
| AGO | 0.005 | 0.002 | 0.001 | 0.006 | 0.005 | 0.0012 |
| BEN | 0.005 | 0.002 | 0.001 | 0.006 | 0.005 | 0.0012 |
| BWA | 0.005 | 0.002 | 0.001 | 0.006 | 0.005 | 0.0012 |
| BFA | 0.005 | 0.002 | 0.001 | 0.006 | 0.005 | 0.0012 |
| BDI | 0.005 | 0.002 | 0.001 | 0.006 | 0.005 | 0.0012 |
| CMR | 0.005 | 0.002 | 0.001 | 0.006 | 0.005 | 0.0012 |
| CPV | 0.005 | 0.002 | 0.001 | 0.006 | 0.005 | 0.0012 |
| CAF | 0.005 | 0.002 | 0.001 | 0.006 | 0.005 | 0.0012 |
| TCD | 0.005 | 0.002 | 0.001 | 0.006 | 0.005 | 0.0012 |
| COM | 0.005 | 0.002 | 0.001 | 0.006 | 0.005 | 0.0012 |
| COG | 0.005 | 0.002 | 0.001 | 0.006 | 0.005 | 0.0012 |
| COD | 0.005 | 0.002 | 0.001 | 0.006 | 0.005 | 0.0012 |
| CIV | 0.005 | 0.002 | 0.001 | 0.006 | 0.005 | 0.0012 |
| DJI | 0.005 | 0.002 | 0.001 | 0.006 | 0.005 | 0.0012 |
| EGY | 0.005 | 0.002 | 0.001 | 0.006 | 0.005 | 0.0012 |
| GNQ | 0.005 | 0.002 | 0.001 | 0.006 | 0.005 | 0.0012 |
| ERI | 0.005 | 0.002 | 0.001 | 0.006 | 0.005 | 0.0012 |
| ETH | 0.005 | 0.002 | 0.001 | 0.006 | 0.005 | 0.0012 |
| GAB | 0.005 | 0.002 | 0.001 | 0.006 | 0.005 | 0.0012 |
| GMB | 0.005 | 0.002 | 0.001 | 0.006 | 0.005 | 0.0012 |
| GHA | 0.005 | 0.002 | 0.001 | 0.006 | 0.005 | 0.0012 |
| GIN | 0.005 | 0.002 | 0.001 | 0.006 | 0.005 | 0.0012 |
| KEN | 0.005 | 0.002 | 0.001 | 0.006 | 0.005 | 0.0012 |
| LSO | 0.005 | 0.002 | 0.001 | 0.006 | 0.005 | 0.0012 |
| LBR | 0.005 | 0.002 | 0.001 | 0.006 | 0.005 | 0.0012 |
| MDG | 0.005 | 0.002 | 0.001 | 0.006 | 0.005 | 0.0012 |
| MWI | 0.005 | 0.002 | 0.001 | 0.006 | 0.005 | 0.0012 |
| MLI | 0.005 | 0.002 | 0.001 | 0.006 | 0.005 | 0.0012 |
| MRT | 0.005 | 0.002 | 0.001 | 0.006 | 0.005 | 0.0012 |
| MUS | 0.005 | 0.002 | 0.001 | 0.006 | 0.005 | 0.0012 |
| MAR | 0.005 | 0.002 | 0.001 | 0.006 | 0.005 | 0.0012 |
| MOZ | 0.005 | 0.002 | 0.001 | 0.006 | 0.005 | 0.0012 |
| NAM | 0.005 | 0.002 | 0.001 | 0.006 | 0.005 | 0.0012 |
| NER | 0.005 | 0.002 | 0.001 | 0.006 | 0.005 | 0.0012 |
| NGA | 0.005 | 0.002 | 0.001 | 0.006 | 0.005 | 0.0012 |
| RWA | 0.005 | 0.002 | 0.001 | 0.006 | 0.005 | 0.0012 |
| SEN | 0.005 | 0.002 | 0.001 | 0.006 | 0.005 | 0.0012 |
| SYC | 0.005 | 0.002 | 0.001 | 0.006 | 0.005 | 0.0012 |
| SLE | 0.005 | 0.002 | 0.001 | 0.006 | 0.005 | 0.0012 |
| ZAF | 0.005 | 0.002 | 0.001 | 0.006 | 0.005 | 0.0012 |
| SSD | 0.005 | 0.002 | 0.001 | 0.006 | 0.005 | 0.0012 |
| SDN | 0.005 | 0.002 | 0.001 | 0.006 | 0.005 | 0.0012 |
| TZA | 0.005 | 0.002 | 0.001 | 0.006 | 0.005 | 0.0012 |
| TGO | 0.005 | 0.002 | 0.001 | 0.006 | 0.005 | 0.0012 |
| TUN | 0.005 | 0.002 | 0.001 | 0.006 | 0.005 | 0.0012 |
| UGA | 0.005 | 0.002 | 0.001 | 0.006 | 0.005 | 0.0012 |
| ZMB | 0.005 | 0.002 | 0.001 | 0.006 | 0.005 | 0.0012 |
| ZWE | 0.005 | 0.002 | 0.001 | 0.006 | 0.005 | 0.0012 |

Table A20. The observational evaluation matrix for 2021 data

| **Country** | **C1** | **C2** | **C3** | **C4** | **C5** | **C6** |
| --- | --- | --- | --- | --- | --- | --- |
| DZA | 0.0016 | 0.0006 | 0.0001 | 0.0057 | 0.0048 | 0.0000 |
| AGO | 0.0025 | 0.0003 | 0.0012 | 0.0032 | 0.0026 | 0.0002 |
| BEN | 0.0001 | 0.0001 | 0.0000 | 0.0004 | 0.0003 | 0.0012 |
| BWA | 0.0001 | 0.0000 | 0.0000 | 0.0002 | 0.0005 | 0.0000 |
| BFA | 0.0000 | 0.0000 | 0.0000 | 0.0000 | 0.0000 | 0.0000 |
| BDI | 0.0000 | 0.0000 | 0.0000 | 0.0002 | 0.0001 | 0.0000 |
| CMR | 0.0004 | 0.0003 | 0.0001 | 0.0015 | 0.0011 | 0.0000 |
| CPV | 0.0000 | 0.0000 | 0.0000 | 0.0000 | 0.0000 | 0.0000 |
| CAF | 0.0000 | 0.0000 | 0.0000 | 0.0001 | 0.0001 | 0.0000 |
| TCD | 0.0006 | 0.0000 | 0.0000 | 0.0010 | 0.0009 | 0.0000 |
| COM | 0.0000 | 0.0000 | 0.0000 | 0.0000 | 0.0000 | 0.0000 |
| COG | 0.0009 | 0.0001 | 0.0003 | 0.0008 | 0.0008 | 0.0000 |
| COD | 0.0039 | 0.0000 | 0.0007 | 0.0039 | 0.0026 | 0.0000 |
| CIV | 0.0007 | 0.0003 | 0.0000 | 0.0013 | 0.0013 | 0.0000 |
| DJI | 0.0001 | 0.0003 | 0.0000 | 0.0003 | 0.0002 | 0.0000 |
| EGY | 0.0012 | 0.0019 | 0.0001 | 0.0032 | 0.0024 | 0.0000 |
| GNQ | 0.0003 | 0.0000 | 0.0001 | 0.0005 | 0.0004 | 0.0000 |
| ERI | 0.0002 | 0.0000 | 0.0000 | 0.0001 | 0.0000 | 0.0000 |
| ETH | 0.0026 | 0.0002 | 0.0000 | 0.0038 | 0.0025 | 0.0000 |
| GAB | 0.0002 | 0.0000 | 0.0001 | 0.0002 | 0.0003 | 0.0000 |
| GMB | 0.0000 | 0.0001 | 0.0000 | 0.0000 | 0.0000 | 0.0000 |
| GHA | 0.0010 | 0.0008 | 0.0001 | 0.0010 | 0.0020 | 0.0000 |
| GIN | 0.0009 | 0.0002 | 0.0002 | 0.0034 | 0.0014 | 0.0000 |
| KEN | 0.0021 | 0.0007 | 0.0000 | 0.0026 | 0.0030 | 0.0000 |
| LSO | 0.0000 | 0.0000 | 0.0000 | 0.0002 | 0.0001 | 0.0000 |
| LBR | 0.0001 | 0.0006 | 0.0000 | 0.0001 | 0.0003 | 0.0000 |
| MDG | 0.0003 | 0.0001 | 0.0000 | 0.0006 | 0.0005 | 0.0000 |
| MWI | 0.0002 | 0.0000 | 0.0000 | 0.0003 | 0.0002 | 0.0000 |
| MLI | 0.0004 | 0.0001 | 0.0000 | 0.0003 | 0.0005 | 0.0000 |
| MRT | 0.0001 | 0.0001 | 0.0001 | 0.0004 | 0.0001 | 0.0000 |
| MUS | 0.0010 | 0.0001 | 0.0000 | 0.0003 | 0.0001 | 0.0000 |
| MAR | 0.0003 | 0.0006 | 0.0000 | 0.0002 | 0.0003 | 0.0000 |
| MOZ | 0.0012 | 0.0003 | 0.0001 | 0.0010 | 0.0010 | 0.0000 |
| NAM | 0.0002 | 0.0000 | 0.0000 | 0.0003 | 0.0004 | 0.0000 |
| NER | 0.0013 | 0.0000 | 0.0000 | 0.0007 | 0.0008 | 0.0000 |
| NGA | 0.0025 | 0.0023 | 0.0002 | 0.0041 | 0.0051 | 0.0000 |
| RWA | 0.0002 | 0.0000 | 0.0000 | 0.0005 | 0.0004 | 0.0000 |
| SEN | 0.0004 | 0.0003 | 0.0000 | 0.0010 | 0.0010 | 0.0008 |
| SYC | 0.0004 | 0.0000 | 0.0000 | 0.0001 | 0.0000 | 0.0000 |
| SLE | 0.0001 | 0.0000 | 0.0000 | 0.0001 | 0.0001 | 0.0000 |
| ZAF | 0.0048 | 0.0022 | 0.0008 | 0.0002 | 0.0010 | 0.0000 |
| SSD | 0.0001 | 0.0000 | 0.0000 | 0.0004 | 0.0003 | 0.0000 |
| SDN | 0.0010 | 0.0002 | 0.0000 | 0.0008 | 0.0004 | 0.0000 |
| TZA | 0.0014 | 0.0006 | 0.0000 | 0.0021 | 0.0021 | 0.0000 |
| TGO | 0.0001 | 0.0003 | 0.0000 | 0.0002 | 0.0001 | 0.0000 |
| TUN | 0.0000 | 0.0002 | 0.0000 | 0.0001 | 0.0001 | 0.0000 |
| UGA | 0.0006 | 0.0001 | 0.0000 | 0.0013 | 0.0013 | 0.0008 |
| ZMB | 0.0028 | 0.0001 | 0.0002 | 0.0024 | 0.0016 | 0.0000 |
| ZWE | 0.0016 | 0.0001 | 0.0001 | 0.0007 | 0.0009 | 0.0000 |

Table A21. The theoretical evaluation matrix for 2022 data

| **Country** | **C1** | **C2** | **C3** | **C4** | **C5** | **C6** |
| --- | --- | --- | --- | --- | --- | --- |
| DZA | 0.004 | 0.003 | 0.001 | 0.007 | 0.005 | 0.0005 |
| AGO | 0.004 | 0.003 | 0.001 | 0.007 | 0.005 | 0.0005 |
| BEN | 0.004 | 0.003 | 0.001 | 0.007 | 0.005 | 0.0005 |
| BWA | 0.004 | 0.003 | 0.001 | 0.007 | 0.005 | 0.0005 |
| BFA | 0.004 | 0.003 | 0.001 | 0.007 | 0.005 | 0.0005 |
| BDI | 0.004 | 0.003 | 0.001 | 0.007 | 0.005 | 0.0005 |
| CMR | 0.004 | 0.003 | 0.001 | 0.007 | 0.005 | 0.0005 |
| CPV | 0.004 | 0.003 | 0.001 | 0.007 | 0.005 | 0.0005 |
| CAF | 0.004 | 0.003 | 0.001 | 0.007 | 0.005 | 0.0005 |
| TCD | 0.004 | 0.003 | 0.001 | 0.007 | 0.005 | 0.0005 |
| COM | 0.004 | 0.003 | 0.001 | 0.007 | 0.005 | 0.0005 |
| COG | 0.004 | 0.003 | 0.001 | 0.007 | 0.005 | 0.0005 |
| COD | 0.004 | 0.003 | 0.001 | 0.007 | 0.005 | 0.0005 |
| CIV | 0.004 | 0.003 | 0.001 | 0.007 | 0.005 | 0.0005 |
| DJI | 0.004 | 0.003 | 0.001 | 0.007 | 0.005 | 0.0005 |
| EGY | 0.004 | 0.003 | 0.001 | 0.007 | 0.005 | 0.0005 |
| GNQ | 0.004 | 0.003 | 0.001 | 0.007 | 0.005 | 0.0005 |
| ERI | 0.004 | 0.003 | 0.001 | 0.007 | 0.005 | 0.0005 |
| ETH | 0.004 | 0.003 | 0.001 | 0.007 | 0.005 | 0.0005 |
| GAB | 0.004 | 0.003 | 0.001 | 0.007 | 0.005 | 0.0005 |
| GMB | 0.004 | 0.003 | 0.001 | 0.007 | 0.005 | 0.0005 |
| GHA | 0.004 | 0.003 | 0.001 | 0.007 | 0.005 | 0.0005 |
| GIN | 0.004 | 0.003 | 0.001 | 0.007 | 0.005 | 0.0005 |
| KEN | 0.004 | 0.003 | 0.001 | 0.007 | 0.005 | 0.0005 |
| LSO | 0.004 | 0.003 | 0.001 | 0.007 | 0.005 | 0.0005 |
| LBR | 0.004 | 0.003 | 0.001 | 0.007 | 0.005 | 0.0005 |
| MDG | 0.004 | 0.003 | 0.001 | 0.007 | 0.005 | 0.0005 |
| MWI | 0.004 | 0.003 | 0.001 | 0.007 | 0.005 | 0.0005 |
| MLI | 0.004 | 0.003 | 0.001 | 0.007 | 0.005 | 0.0005 |
| MRT | 0.004 | 0.003 | 0.001 | 0.007 | 0.005 | 0.0005 |
| MUS | 0.004 | 0.003 | 0.001 | 0.007 | 0.005 | 0.0005 |
| MAR | 0.004 | 0.003 | 0.001 | 0.007 | 0.005 | 0.0005 |
| MOZ | 0.004 | 0.003 | 0.001 | 0.007 | 0.005 | 0.0005 |
| NAM | 0.004 | 0.003 | 0.001 | 0.007 | 0.005 | 0.0005 |
| NER | 0.004 | 0.003 | 0.001 | 0.007 | 0.005 | 0.0005 |
| NGA | 0.004 | 0.003 | 0.001 | 0.007 | 0.005 | 0.0005 |
| RWA | 0.004 | 0.003 | 0.001 | 0.007 | 0.005 | 0.0005 |
| SEN | 0.004 | 0.003 | 0.001 | 0.007 | 0.005 | 0.0005 |
| SYC | 0.004 | 0.003 | 0.001 | 0.007 | 0.005 | 0.0005 |
| SLE | 0.004 | 0.003 | 0.001 | 0.007 | 0.005 | 0.0005 |
| ZAF | 0.004 | 0.003 | 0.001 | 0.007 | 0.005 | 0.0005 |
| SSD | 0.004 | 0.003 | 0.001 | 0.007 | 0.005 | 0.0005 |
| SDN | 0.004 | 0.003 | 0.001 | 0.007 | 0.005 | 0.0005 |
| TZA | 0.004 | 0.003 | 0.001 | 0.007 | 0.005 | 0.0005 |
| TGO | 0.004 | 0.003 | 0.001 | 0.007 | 0.005 | 0.0005 |
| TUN | 0.004 | 0.003 | 0.001 | 0.007 | 0.005 | 0.0005 |
| UGA | 0.004 | 0.003 | 0.001 | 0.007 | 0.005 | 0.0005 |
| ZMB | 0.004 | 0.003 | 0.001 | 0.007 | 0.005 | 0.0005 |
| ZWE | 0.004 | 0.003 | 0.001 | 0.007 | 0.005 | 0.0005 |

Table A22. The observational evaluation matrix for 2022 data

| **Country** | **C1** | **C2** | **C3** | **C4** | **C5** | **C6** |
| --- | --- | --- | --- | --- | --- | --- |
| DZA | 0.0011 | 0.0007 | 0.0001 | 0.0063 | 0.0031 | 0.0000 |
| AGO | 0.0013 | 0.0004 | 0.0014 | 0.0036 | 0.0032 | 0.0000 |
| BEN | 0.0001 | 0.0002 | 0.0000 | 0.0013 | 0.0007 | 0.0000 |
| BWA | 0.0001 | 0.0000 | 0.0000 | 0.0003 | 0.0005 | 0.0000 |
| BFA | 0.0000 | 0.0000 | 0.0000 | 0.0004 | 0.0001 | 0.0000 |
| BDI | 0.0000 | 0.0000 | 0.0000 | 0.0002 | 0.0000 | 0.0000 |
| CMR | 0.0003 | 0.0003 | 0.0000 | 0.0020 | 0.0007 | 0.0000 |
| CPV | 0.0000 | 0.0000 | 0.0000 | 0.0000 | 0.0000 | 0.0000 |
| CAF | 0.0000 | 0.0000 | 0.0000 | 0.0002 | 0.0001 | 0.0000 |
| TCD | 0.0004 | 0.0000 | 0.0001 | 0.0013 | 0.0007 | 0.0000 |
| COM | 0.0000 | 0.0000 | 0.0000 | 0.0001 | 0.0000 | 0.0000 |
| COG | 0.0003 | 0.0001 | 0.0003 | 0.0009 | 0.0003 | 0.0000 |
| COD | 0.0027 | 0.0006 | 0.0010 | 0.0054 | 0.0028 | 0.0000 |
| CIV | 0.0005 | 0.0004 | 0.0001 | 0.0026 | 0.0017 | 0.0002 |
| DJI | 0.0001 | 0.0004 | 0.0000 | 0.0004 | 0.0002 | 0.0000 |
| EGY | 0.0008 | 0.0019 | 0.0001 | 0.0072 | 0.0030 | 0.0000 |
| GNQ | 0.0002 | 0.0000 | 0.0001 | 0.0007 | 0.0004 | 0.0000 |
| ERI | 0.0002 | 0.0000 | 0.0000 | 0.0006 | 0.0001 | 0.0000 |
| ETH | 0.0017 | 0.0002 | 0.0000 | 0.0041 | 0.0017 | 0.0000 |
| GAB | 0.0001 | 0.0001 | 0.0002 | 0.0002 | 0.0002 | 0.0000 |
| GMB | 0.0000 | 0.0000 | 0.0000 | 0.0000 | 0.0000 | 0.0000 |
| GHA | 0.0007 | 0.0009 | 0.0001 | 0.0017 | 0.0014 | 0.0001 |
| GIN | 0.0007 | 0.0002 | 0.0003 | 0.0050 | 0.0014 | 0.0000 |
| KEN | 0.0012 | 0.0009 | 0.0000 | 0.0040 | 0.0023 | 0.0000 |
| LSO | 0.0000 | 0.0000 | 0.0000 | 0.0003 | 0.0001 | 0.0000 |
| LBR | 0.0001 | 0.0008 | 0.0000 | 0.0003 | 0.0001 | 0.0000 |
| MDG | 0.0002 | 0.0002 | 0.0000 | 0.0004 | 0.0003 | 0.0000 |
| MWI | 0.0001 | 0.0000 | 0.0000 | 0.0004 | 0.0005 | 0.0000 |
| MLI | 0.0003 | 0.0001 | 0.0000 | 0.0002 | 0.0005 | 0.0000 |
| MRT | 0.0001 | 0.0001 | 0.0001 | 0.0003 | 0.0000 | 0.0000 |
| MUS | 0.0010 | 0.0001 | 0.0000 | 0.0003 | 0.0002 | 0.0000 |
| MAR | 0.0002 | 0.0006 | 0.0001 | 0.0002 | 0.0003 | 0.0000 |
| MOZ | 0.0008 | 0.0004 | 0.0001 | 0.0011 | 0.0008 | 0.0000 |
| NAM | 0.0001 | 0.0001 | 0.0000 | 0.0004 | 0.0003 | 0.0000 |
| NER | 0.0012 | 0.0001 | 0.0000 | 0.0022 | 0.0011 | 0.0000 |
| NGA | 0.0015 | 0.0024 | 0.0001 | 0.0053 | 0.0050 | 0.0000 |
| RWA | 0.0001 | 0.0000 | 0.0000 | 0.0007 | 0.0004 | 0.0000 |
| SEN | 0.0001 | 0.0004 | 0.0000 | 0.0014 | 0.0009 | 0.0005 |
| SYC | 0.0003 | 0.0000 | 0.0000 | 0.0001 | 0.0000 | 0.0000 |
| SLE | 0.0001 | 0.0001 | 0.0000 | 0.0002 | 0.0001 | 0.0000 |
| ZAF | 0.0037 | 0.0026 | 0.0007 | 0.0005 | 0.0007 | 0.0000 |
| SSD | 0.0000 | 0.0000 | 0.0000 | 0.0003 | 0.0002 | 0.0000 |
| SDN | 0.0006 | 0.0002 | 0.0001 | 0.0009 | 0.0004 | 0.0000 |
| TZA | 0.0009 | 0.0008 | 0.0000 | 0.0028 | 0.0014 | 0.0000 |
| TGO | 0.0000 | 0.0003 | 0.0000 | 0.0002 | 0.0001 | 0.0000 |
| TUN | 0.0000 | 0.0002 | 0.0000 | 0.0002 | 0.0001 | 0.0000 |
| UGA | 0.0005 | 0.0001 | 0.0000 | 0.0019 | 0.0012 | 0.0001 |
| ZMB | 0.0013 | 0.0001 | 0.0003 | 0.0035 | 0.0013 | 0.0000 |
| ZWE | 0.0010 | 0.0001 | 0.0001 | 0.0011 | 0.0006 | 0.0000 |

Table A23. The total gap matrix for 2018 data

| **Country** | **C1** | **C2** | **C3** | **C4** | **C5** | **C6** |
| --- | --- | --- | --- | --- | --- | --- |
| DZA | 0.003 | 0.002 | 0.001 | 0.000 | 0.000 | 0.0060 |
| AGO | 0.003 | 0.003 | 0.000 | 0.000 | 0.002 | 0.0022 |
| BEN | 0.005 | 0.003 | 0.001 | 0.000 | 0.005 | 0.0060 |
| BWA | 0.005 | 0.003 | 0.001 | 0.000 | 0.005 | 0.0060 |
| BFA | 0.005 | 0.003 | 0.001 | 0.000 | 0.005 | 0.0060 |
| BDI | 0.005 | 0.004 | 0.001 | 0.000 | 0.005 | 0.0060 |
| CMR | 0.005 | 0.003 | 0.001 | 0.000 | 0.004 | 0.0055 |
| CPV | 0.005 | 0.004 | 0.001 | 0.000 | 0.005 | 0.0060 |
| CAF | 0.005 | 0.004 | 0.001 | 0.000 | 0.005 | 0.0060 |
| TCD | 0.004 | 0.003 | 0.001 | 0.000 | 0.005 | 0.0060 |
| COM | 0.005 | 0.004 | 0.001 | 0.000 | 0.005 | 0.0057 |
| COG | 0.004 | 0.003 | 0.000 | 0.000 | 0.005 | 0.0060 |
| COD | 0.002 | 0.003 | 0.000 | 0.000 | 0.004 | 0.0039 |
| CIV | 0.005 | 0.003 | 0.001 | 0.000 | 0.004 | 0.0051 |
| DJI | 0.005 | 0.003 | 0.001 | 0.000 | 0.005 | 0.0060 |
| EGY | 0.004 | 0.001 | 0.001 | 0.000 | 0.004 | 0.0006 |
| GNQ | 0.004 | 0.004 | 0.001 | 0.000 | 0.005 | 0.0060 |
| ERI | 0.005 | 0.004 | 0.001 | 0.000 | 0.005 | 0.0057 |
| ETH | 0.003 | 0.003 | 0.001 | 0.000 | 0.002 | 0.0053 |
| GAB | 0.005 | 0.003 | 0.000 | 0.000 | 0.005 | 0.0058 |
| GMB | 0.005 | 0.003 | 0.001 | 0.000 | 0.005 | 0.0059 |
| GHA | 0.004 | 0.002 | 0.001 | 0.000 | 0.004 | 0.0040 |
| GIN | 0.004 | 0.003 | 0.001 | 0.000 | 0.004 | 0.0001 |
| KEN | 0.004 | 0.002 | 0.001 | 0.000 | 0.002 | 0.0055 |
| LSO | 0.005 | 0.004 | 0.001 | 0.000 | 0.005 | 0.0057 |
| LBR | 0.005 | 0.003 | 0.001 | 0.000 | 0.005 | 0.0060 |
| MDG | 0.004 | 0.003 | 0.001 | 0.000 | 0.005 | 0.0057 |
| MWI | 0.005 | 0.003 | 0.001 | 0.000 | 0.005 | 0.0060 |
| MLI | 0.005 | 0.003 | 0.001 | 0.000 | 0.005 | 0.0055 |
| MRT | 0.005 | 0.003 | 0.001 | 0.000 | 0.005 | 0.0060 |
| MUS | 0.004 | 0.003 | 0.001 | 0.000 | 0.005 | 0.0060 |
| MAR | 0.005 | 0.003 | 0.001 | 0.000 | 0.004 | 0.0055 |
| MOZ | 0.004 | 0.003 | 0.001 | 0.000 | 0.004 | 0.0060 |
| NAM | 0.005 | 0.003 | 0.001 | 0.000 | 0.005 | 0.0059 |
| NER | 0.004 | 0.004 | 0.001 | 0.000 | 0.005 | 0.0060 |
| NGA | 0.003 | 0.001 | 0.001 | 0.000 | 0.002 | 0.0034 |
| RWA | 0.005 | 0.003 | 0.001 | 0.000 | 0.005 | 0.0056 |
| SEN | 0.005 | 0.003 | 0.001 | 0.000 | 0.004 | 0.0034 |
| SYC | 0.005 | 0.004 | 0.001 | 0.000 | 0.005 | 0.0060 |
| SLE | 0.005 | 0.003 | 0.001 | 0.000 | 0.005 | 0.0060 |
| ZAF | 0.000 | 0.000 | 0.000 | 0.000 | 0.004 | 0.0000 |
| SSD | 0.005 | 0.004 | 0.001 | 0.000 | 0.005 | 0.0052 |
| SDN | 0.004 | 0.003 | 0.001 | 0.000 | 0.005 | 0.0059 |
| TZA | 0.004 | 0.003 | 0.001 | 0.000 | 0.004 | 0.0060 |
| TGO | 0.005 | 0.003 | 0.001 | 0.000 | 0.005 | 0.0060 |
| TUN | 0.005 | 0.003 | 0.001 | 0.000 | 0.005 | 0.0060 |
| UGA | 0.004 | 0.003 | 0.001 | 0.000 | 0.004 | 0.0060 |
| ZMB | 0.002 | 0.003 | 0.000 | 0.000 | 0.003 | 0.0030 |
| ZWE | 0.004 | 0.003 | 0.001 | 0.000 | 0.005 | 0.0055 |

Table A24. The total gap matrix for 2019 data

| **Country** | **C1** | **C2** | **C3** | **C4** | **C5** | **C6** |
| --- | --- | --- | --- | --- | --- | --- |
| DZA | 0.004 | 0.003 | 0.001 | 0.000 | 0.000 | 0.0002 |
| AGO | 0.003 | 0.004 | 0.000 | 0.001 | 0.004 | 0.0002 |
| BEN | 0.006 | 0.004 | 0.001 | 0.001 | 0.007 | 0.0002 |
| BWA | 0.006 | 0.004 | 0.001 | 0.001 | 0.007 | 0.0002 |
| BFA | 0.006 | 0.004 | 0.001 | 0.001 | 0.007 | 0.0002 |
| BDI | 0.006 | 0.004 | 0.001 | 0.001 | 0.007 | 0.0002 |
| CMR | 0.006 | 0.004 | 0.001 | 0.001 | 0.006 | 0.0002 |
| CPV | 0.006 | 0.004 | 0.001 | 0.001 | 0.007 | 0.0002 |
| CAF | 0.006 | 0.004 | 0.001 | 0.001 | 0.007 | 0.0002 |
| TCD | 0.006 | 0.004 | 0.001 | 0.001 | 0.007 | 0.0002 |
| COM | 0.006 | 0.004 | 0.001 | 0.001 | 0.007 | 0.0002 |
| COG | 0.006 | 0.004 | 0.001 | 0.001 | 0.007 | 0.0002 |
| COD | 0.001 | 0.004 | 0.001 | 0.001 | 0.005 | 0.0002 |
| CIV | 0.006 | 0.004 | 0.001 | 0.001 | 0.006 | 0.0002 |
| DJI | 0.006 | 0.004 | 0.001 | 0.001 | 0.007 | 0.0002 |
| EGY | 0.005 | 0.001 | 0.001 | 0.001 | 0.004 | 0.0000 |
| GNQ | 0.006 | 0.004 | 0.001 | 0.001 | 0.007 | 0.0002 |
| ERI | 0.006 | 0.004 | 0.001 | 0.001 | 0.007 | 0.0002 |
| ETH | 0.004 | 0.004 | 0.001 | 0.001 | 0.005 | 0.0002 |
| GAB | 0.006 | 0.004 | 0.001 | 0.001 | 0.007 | 0.0002 |
| GMB | 0.006 | 0.004 | 0.001 | 0.001 | 0.007 | 0.0002 |
| GHA | 0.004 | 0.003 | 0.001 | 0.001 | 0.006 | 0.0002 |
| GIN | 0.005 | 0.004 | 0.001 | 0.001 | 0.006 | 0.0002 |
| KEN | 0.005 | 0.003 | 0.001 | 0.001 | 0.003 | 0.0002 |
| LSO | 0.006 | 0.004 | 0.001 | 0.001 | 0.007 | 0.0002 |
| LBR | 0.006 | 0.003 | 0.001 | 0.001 | 0.007 | 0.0002 |
| MDG | 0.006 | 0.004 | 0.001 | 0.001 | 0.007 | 0.0002 |
| MWI | 0.006 | 0.004 | 0.001 | 0.001 | 0.007 | 0.0002 |
| MLI | 0.006 | 0.004 | 0.001 | 0.001 | 0.007 | 0.0002 |
| MRT | 0.006 | 0.004 | 0.001 | 0.001 | 0.007 | 0.0002 |
| MUS | 0.005 | 0.004 | 0.001 | 0.001 | 0.007 | 0.0002 |
| MAR | 0.006 | 0.003 | 0.001 | 0.001 | 0.007 | 0.0002 |
| MOZ | 0.005 | 0.004 | 0.001 | 0.001 | 0.006 | 0.0002 |
| NAM | 0.006 | 0.004 | 0.001 | 0.001 | 0.007 | 0.0002 |
| NER | 0.005 | 0.004 | 0.001 | 0.001 | 0.007 | 0.0002 |
| NGA | 0.004 | 0.000 | 0.001 | 0.001 | 0.002 | 0.0002 |
| RWA | 0.006 | 0.004 | 0.001 | 0.001 | 0.007 | 0.0002 |
| SEN | 0.006 | 0.004 | 0.001 | 0.001 | 0.006 | 0.0002 |
| SYC | 0.006 | 0.004 | 0.001 | 0.001 | 0.007 | 0.0002 |
| SLE | 0.006 | 0.004 | 0.001 | 0.001 | 0.007 | 0.0002 |
| ZAF | 0.000 | 0.000 | 0.001 | 0.001 | 0.007 | 0.0002 |
| SSD | 0.006 | 0.004 | 0.001 | 0.001 | 0.007 | 0.0002 |
| SDN | 0.005 | 0.004 | 0.001 | 0.001 | 0.007 | 0.0002 |
| TZA | 0.005 | 0.003 | 0.001 | 0.001 | 0.006 | 0.0002 |
| TGO | 0.006 | 0.004 | 0.001 | 0.001 | 0.007 | 0.0002 |
| TUN | 0.006 | 0.004 | 0.001 | 0.001 | 0.007 | 0.0002 |
| UGA | 0.006 | 0.004 | 0.001 | 0.001 | 0.006 | 0.0002 |
| ZMB | 0.003 | 0.004 | 0.001 | 0.001 | 0.004 | 0.0002 |
| ZWE | 0.004 | 0.004 | 0.001 | 0.001 | 0.007 | 0.0002 |

Table A25. The total gap matrix for 2020 data

| **Country** | **C1** | **C2** | **C3** | **C4** | **C5** | **C6** |
| --- | --- | --- | --- | --- | --- | --- |
| DZA | 0.004 | 0.002 | 0.001 | 0.000 | 0.000 | 0.0015 |
| AGO | 0.003 | 0.003 | 0.000 | 0.002 | 0.004 | 0.0015 |
| BEN | 0.005 | 0.003 | 0.001 | 0.003 | 0.005 | 0.0015 |
| BWA | 0.005 | 0.003 | 0.001 | 0.003 | 0.005 | 0.0015 |
| BFA | 0.006 | 0.003 | 0.001 | 0.003 | 0.006 | 0.0013 |
| BDI | 0.006 | 0.003 | 0.001 | 0.003 | 0.006 | 0.0015 |
| CMR | 0.005 | 0.002 | 0.001 | 0.003 | 0.005 | 0.0015 |
| CPV | 0.006 | 0.003 | 0.001 | 0.003 | 0.006 | 0.0015 |
| CAF | 0.006 | 0.003 | 0.001 | 0.003 | 0.006 | 0.0015 |
| TCD | 0.005 | 0.003 | 0.001 | 0.003 | 0.005 | 0.0015 |
| COM | 0.006 | 0.003 | 0.001 | 0.003 | 0.006 | 0.0015 |
| COG | 0.004 | 0.003 | 0.001 | 0.003 | 0.005 | 0.0015 |
| COD | 0.002 | 0.002 | 0.001 | 0.002 | 0.003 | 0.0011 |
| CIV | 0.005 | 0.002 | 0.001 | 0.003 | 0.005 | 0.0014 |
| DJI | 0.005 | 0.002 | 0.001 | 0.003 | 0.006 | 0.0015 |
| EGY | 0.004 | 0.001 | 0.001 | 0.003 | 0.002 | 0.0015 |
| GNQ | 0.005 | 0.003 | 0.001 | 0.003 | 0.005 | 0.0015 |
| ERI | 0.005 | 0.003 | 0.001 | 0.003 | 0.006 | 0.0015 |
| ETH | 0.002 | 0.002 | 0.001 | 0.001 | 0.002 | 0.0015 |
| GAB | 0.005 | 0.003 | 0.001 | 0.003 | 0.006 | 0.0015 |
| GMB | 0.005 | 0.003 | 0.001 | 0.003 | 0.006 | 0.0015 |
| GHA | 0.004 | 0.002 | 0.001 | 0.003 | 0.004 | 0.0000 |
| GIN | 0.005 | 0.002 | 0.001 | 0.002 | 0.004 | 0.0015 |
| KEN | 0.003 | 0.002 | 0.001 | 0.002 | 0.002 | 0.0015 |
| LSO | 0.005 | 0.003 | 0.001 | 0.003 | 0.006 | 0.0013 |
| LBR | 0.005 | 0.002 | 0.001 | 0.003 | 0.005 | 0.0015 |
| MDG | 0.005 | 0.003 | 0.001 | 0.003 | 0.005 | 0.0014 |
| MWI | 0.005 | 0.003 | 0.001 | 0.003 | 0.006 | 0.0012 |
| MLI | 0.005 | 0.003 | 0.001 | 0.003 | 0.005 | 0.0015 |
| MRT | 0.005 | 0.003 | 0.001 | 0.003 | 0.006 | 0.0015 |
| MUS | 0.005 | 0.003 | 0.001 | 0.003 | 0.005 | 0.0015 |
| MAR | 0.005 | 0.002 | 0.001 | 0.003 | 0.005 | 0.0015 |
| MOZ | 0.004 | 0.002 | 0.001 | 0.003 | 0.005 | 0.0011 |
| NAM | 0.005 | 0.003 | 0.001 | 0.003 | 0.005 | 0.0015 |
| NER | 0.004 | 0.003 | 0.001 | 0.003 | 0.005 | 0.0015 |
| NGA | 0.003 | 0.000 | 0.001 | 0.002 | 0.001 | 0.0015 |
| RWA | 0.005 | 0.003 | 0.001 | 0.003 | 0.005 | 0.0009 |
| SEN | 0.005 | 0.002 | 0.001 | 0.003 | 0.005 | 0.0015 |
| SYC | 0.005 | 0.003 | 0.001 | 0.003 | 0.006 | 0.0015 |
| SLE | 0.005 | 0.003 | 0.001 | 0.003 | 0.006 | 0.0015 |
| ZAF | 0.000 | 0.000 | 0.000 | 0.003 | 0.005 | 0.0015 |
| SSD | 0.005 | 0.003 | 0.001 | 0.003 | 0.005 | 0.0015 |
| SDN | 0.004 | 0.002 | 0.001 | 0.003 | 0.005 | 0.0015 |
| TZA | 0.004 | 0.002 | 0.001 | 0.003 | 0.004 | 0.0015 |
| TGO | 0.005 | 0.002 | 0.001 | 0.003 | 0.006 | 0.0015 |
| TUN | 0.005 | 0.003 | 0.001 | 0.003 | 0.006 | 0.0015 |
| UGA | 0.005 | 0.003 | 0.001 | 0.003 | 0.004 | 0.0006 |
| ZMB | 0.002 | 0.003 | 0.001 | 0.002 | 0.003 | 0.0012 |
| ZWE | 0.004 | 0.003 | 0.001 | 0.003 | 0.005 | 0.0015 |

Table A26. The total gap matrix for 2021 data

| **Country** | **C1** | **C2** | **C3** | **C4** | **C5** | **C6** |
| --- | --- | --- | --- | --- | --- | --- |
| DZA | 0.003 | 0.002 | 0.001 | 0.000 | 0.000 | 0.0012 |
| AGO | 0.002 | 0.002 | 0.000 | 0.003 | 0.003 | 0.0010 |
| BEN | 0.005 | 0.002 | 0.001 | 0.005 | 0.005 | 0.0000 |
| BWA | 0.005 | 0.002 | 0.001 | 0.006 | 0.005 | 0.0012 |
| BFA | 0.005 | 0.002 | 0.001 | 0.006 | 0.005 | 0.0012 |
| BDI | 0.005 | 0.002 | 0.001 | 0.006 | 0.005 | 0.0012 |
| CMR | 0.004 | 0.002 | 0.001 | 0.004 | 0.004 | 0.0012 |
| CPV | 0.005 | 0.002 | 0.001 | 0.006 | 0.005 | 0.0012 |
| CAF | 0.005 | 0.002 | 0.001 | 0.006 | 0.005 | 0.0012 |
| TCD | 0.004 | 0.002 | 0.001 | 0.005 | 0.004 | 0.0012 |
| COM | 0.005 | 0.002 | 0.001 | 0.006 | 0.005 | 0.0012 |
| COG | 0.004 | 0.002 | 0.001 | 0.005 | 0.004 | 0.0012 |
| COD | 0.001 | 0.002 | 0.001 | 0.002 | 0.003 | 0.0012 |
| CIV | 0.004 | 0.002 | 0.001 | 0.004 | 0.004 | 0.0012 |
| DJI | 0.005 | 0.002 | 0.001 | 0.005 | 0.005 | 0.0012 |
| EGY | 0.004 | 0.000 | 0.001 | 0.003 | 0.003 | 0.0012 |
| GNQ | 0.005 | 0.002 | 0.001 | 0.005 | 0.005 | 0.0012 |
| ERI | 0.005 | 0.002 | 0.001 | 0.006 | 0.005 | 0.0012 |
| ETH | 0.002 | 0.002 | 0.001 | 0.002 | 0.003 | 0.0012 |
| GAB | 0.005 | 0.002 | 0.001 | 0.006 | 0.005 | 0.0012 |
| GMB | 0.005 | 0.002 | 0.001 | 0.006 | 0.005 | 0.0012 |
| GHA | 0.004 | 0.001 | 0.001 | 0.005 | 0.003 | 0.0012 |
| GIN | 0.004 | 0.002 | 0.001 | 0.002 | 0.004 | 0.0012 |
| KEN | 0.003 | 0.002 | 0.001 | 0.003 | 0.002 | 0.0012 |
| LSO | 0.005 | 0.002 | 0.001 | 0.006 | 0.005 | 0.0012 |
| LBR | 0.005 | 0.002 | 0.001 | 0.006 | 0.005 | 0.0012 |
| MDG | 0.005 | 0.002 | 0.001 | 0.005 | 0.005 | 0.0012 |
| MWI | 0.005 | 0.002 | 0.001 | 0.005 | 0.005 | 0.0012 |
| MLI | 0.004 | 0.002 | 0.001 | 0.005 | 0.005 | 0.0012 |
| MRT | 0.005 | 0.002 | 0.001 | 0.005 | 0.005 | 0.0012 |
| MUS | 0.004 | 0.002 | 0.001 | 0.005 | 0.005 | 0.0012 |
| MAR | 0.005 | 0.002 | 0.001 | 0.006 | 0.005 | 0.0012 |
| MOZ | 0.004 | 0.002 | 0.001 | 0.005 | 0.004 | 0.0012 |
| NAM | 0.005 | 0.002 | 0.001 | 0.005 | 0.005 | 0.0012 |
| NER | 0.004 | 0.002 | 0.001 | 0.005 | 0.004 | 0.0012 |
| NGA | 0.002 | 0.000 | 0.001 | 0.002 | 0.000 | 0.0012 |
| RWA | 0.005 | 0.002 | 0.001 | 0.005 | 0.005 | 0.0012 |
| SEN | 0.004 | 0.002 | 0.001 | 0.005 | 0.004 | 0.0004 |
| SYC | 0.004 | 0.002 | 0.001 | 0.006 | 0.005 | 0.0012 |
| SLE | 0.005 | 0.002 | 0.001 | 0.006 | 0.005 | 0.0012 |
| ZAF | 0.000 | 0.000 | 0.000 | 0.006 | 0.004 | 0.0012 |
| SSD | 0.005 | 0.002 | 0.001 | 0.005 | 0.005 | 0.0012 |
| SDN | 0.004 | 0.002 | 0.001 | 0.005 | 0.005 | 0.0012 |
| TZA | 0.003 | 0.002 | 0.001 | 0.004 | 0.003 | 0.0012 |
| TGO | 0.005 | 0.002 | 0.001 | 0.006 | 0.005 | 0.0012 |
| TUN | 0.005 | 0.002 | 0.001 | 0.006 | 0.005 | 0.0012 |
| UGA | 0.004 | 0.002 | 0.001 | 0.004 | 0.004 | 0.0004 |
| ZMB | 0.002 | 0.002 | 0.001 | 0.003 | 0.004 | 0.0012 |
| ZWE | 0.003 | 0.002 | 0.001 | 0.005 | 0.004 | 0.0012 |

Table A27. The total gap matrix for 2022 data

| **Country** | **C1** | **C2** | **C3** | **C4** | **C5** | **C6** |
| --- | --- | --- | --- | --- | --- | --- |
| DZA | 0.003 | 0.002 | 0.001 | 0.001 | 0.002 | 0.0005 |
| AGO | 0.002 | 0.002 | 0.000 | 0.004 | 0.002 | 0.0004 |
| BEN | 0.004 | 0.002 | 0.001 | 0.006 | 0.004 | 0.0004 |
| BWA | 0.004 | 0.003 | 0.001 | 0.007 | 0.004 | 0.0005 |
| BFA | 0.004 | 0.003 | 0.001 | 0.007 | 0.005 | 0.0005 |
| BDI | 0.004 | 0.003 | 0.001 | 0.007 | 0.005 | 0.0005 |
| CMR | 0.003 | 0.002 | 0.001 | 0.005 | 0.004 | 0.0005 |
| CPV | 0.004 | 0.003 | 0.001 | 0.007 | 0.005 | 0.0005 |
| CAF | 0.004 | 0.003 | 0.001 | 0.007 | 0.005 | 0.0005 |
| TCD | 0.003 | 0.003 | 0.001 | 0.006 | 0.004 | 0.0005 |
| COM | 0.004 | 0.003 | 0.001 | 0.007 | 0.005 | 0.0005 |
| COG | 0.003 | 0.003 | 0.001 | 0.006 | 0.005 | 0.0005 |
| COD | 0.001 | 0.002 | 0.000 | 0.002 | 0.002 | 0.0004 |
| CIV | 0.003 | 0.002 | 0.001 | 0.005 | 0.003 | 0.0003 |
| DJI | 0.004 | 0.002 | 0.001 | 0.007 | 0.005 | 0.0005 |
| EGY | 0.003 | 0.001 | 0.001 | 0.000 | 0.002 | 0.0005 |
| GNQ | 0.004 | 0.003 | 0.001 | 0.006 | 0.005 | 0.0005 |
| ERI | 0.004 | 0.003 | 0.001 | 0.007 | 0.005 | 0.0005 |
| ETH | 0.002 | 0.002 | 0.001 | 0.003 | 0.003 | 0.0005 |
| GAB | 0.004 | 0.003 | 0.001 | 0.007 | 0.005 | 0.0005 |
| GMB | 0.004 | 0.003 | 0.001 | 0.007 | 0.005 | 0.0005 |
| GHA | 0.003 | 0.002 | 0.001 | 0.005 | 0.004 | 0.0004 |
| GIN | 0.003 | 0.002 | 0.001 | 0.002 | 0.004 | 0.0005 |
| KEN | 0.003 | 0.002 | 0.001 | 0.003 | 0.003 | 0.0005 |
| LSO | 0.004 | 0.003 | 0.001 | 0.007 | 0.005 | 0.0005 |
| LBR | 0.004 | 0.002 | 0.001 | 0.007 | 0.005 | 0.0005 |
| MDG | 0.004 | 0.002 | 0.001 | 0.007 | 0.005 | 0.0005 |
| MWI | 0.004 | 0.003 | 0.001 | 0.007 | 0.005 | 0.0005 |
| MLI | 0.003 | 0.003 | 0.001 | 0.007 | 0.005 | 0.0005 |
| MRT | 0.004 | 0.003 | 0.001 | 0.007 | 0.005 | 0.0005 |
| MUS | 0.003 | 0.003 | 0.001 | 0.007 | 0.005 | 0.0005 |
| MAR | 0.004 | 0.002 | 0.001 | 0.007 | 0.005 | 0.0005 |
| MOZ | 0.003 | 0.002 | 0.001 | 0.006 | 0.004 | 0.0005 |
| NAM | 0.004 | 0.003 | 0.001 | 0.007 | 0.005 | 0.0005 |
| NER | 0.003 | 0.003 | 0.001 | 0.005 | 0.004 | 0.0005 |
| NGA | 0.002 | 0.000 | 0.001 | 0.002 | 0.000 | 0.0005 |
| RWA | 0.004 | 0.003 | 0.001 | 0.007 | 0.005 | 0.0004 |
| SEN | 0.004 | 0.002 | 0.001 | 0.006 | 0.004 | 0.0000 |
| SYC | 0.003 | 0.003 | 0.001 | 0.007 | 0.005 | 0.0005 |
| SLE | 0.004 | 0.003 | 0.001 | 0.007 | 0.005 | 0.0005 |
| ZAF | 0.000 | 0.000 | 0.001 | 0.007 | 0.004 | 0.0005 |
| SSD | 0.004 | 0.003 | 0.001 | 0.007 | 0.005 | 0.0005 |
| SDN | 0.003 | 0.002 | 0.001 | 0.006 | 0.005 | 0.0005 |
| TZA | 0.003 | 0.002 | 0.001 | 0.004 | 0.004 | 0.0005 |
| TGO | 0.004 | 0.002 | 0.001 | 0.007 | 0.005 | 0.0005 |
| TUN | 0.004 | 0.002 | 0.001 | 0.007 | 0.005 | 0.0005 |
| UGA | 0.003 | 0.003 | 0.001 | 0.005 | 0.004 | 0.0003 |
| ZMB | 0.002 | 0.003 | 0.001 | 0.004 | 0.004 | 0.0005 |
| ZWE | 0.003 | 0.003 | 0.001 | 0.006 | 0.004 | 0.0005 |

Table A28. The total gap and rank of Africa countries in 2019-2022

| **Country** | **Total gap** | | | | | **Rank** | | | | |
| --- | --- | --- | --- | --- | --- | --- | --- | --- | --- | --- |
|  | **2018** | **2019** | **2020** | **2021** | **2022** | **2018** | **2019** | **2020** | **2021** | **2022** |
| DZA | 0.012 | 0.008 | 0.009 | 0.008 | 0.009 | 5 | 2 | 1 | 2 | 4 |
| AGO | 0.011 | 0.012 | 0.013 | 0.010 | 0.011 | 4 | 7 | 7 | 4 | 5 |
| BEN | 0.020 | 0.019 | 0.020 | 0.018 | 0.018 | 34 | 27 | 35 | 23 | 21 |
| BWA | 0.020 | 0.020 | 0.020 | 0.020 | 0.019 | 38 | 37 | 38 | 33 | 32 |
| BFA | 0.020 | 0.020 | 0.020 | 0.020 | 0.020 | 47 | 48 | 44 | 46 | 41 |
| BDI | 0.020 | 0.020 | 0.020 | 0.020 | 0.020 | 48 | 44 | 46 | 44 | 45 |
| CMR | 0.018 | 0.018 | 0.018 | 0.017 | 0.017 | 17 | 17 | 18 | 18 | 16 |
| CPV | 0.020 | 0.020 | 0.020 | 0.020 | 0.020 | 49 | 47 | 49 | 49 | 49 |
| CAF | 0.020 | 0.020 | 0.020 | 0.020 | 0.020 | 46 | 45 | 47 | 45 | 46 |
| TCD | 0.020 | 0.019 | 0.019 | 0.018 | 0.018 | 27 | 22 | 21 | 21 | 20 |
| COM | 0.020 | 0.020 | 0.020 | 0.020 | 0.020 | 43 | 49 | 48 | 48 | 47 |
| COG | 0.019 | 0.019 | 0.018 | 0.018 | 0.019 | 22 | 24 | 17 | 20 | 23 |
| COD | 0.013 | 0.011 | 0.011 | 0.009 | 0.008 | 8 | 4 | 4 | 3 | 3 |
| CIV | 0.018 | 0.018 | 0.018 | 0.017 | 0.015 | 15 | 16 | 16 | 14 | 12 |
| DJI | 0.020 | 0.019 | 0.020 | 0.020 | 0.019 | 29 | 28 | 33 | 35 | 33 |
| EGY | 0.010 | 0.012 | 0.013 | 0.012 | 0.008 | 2 | 5 | 8 | 7 | 2 |
| GNQ | 0.020 | 0.019 | 0.019 | 0.019 | 0.019 | 26 | 26 | 26 | 28 | 26 |
| ERI | 0.020 | 0.020 | 0.020 | 0.020 | 0.020 | 37 | 42 | 43 | 42 | 35 |
| ETH | 0.014 | 0.014 | 0.011 | 0.011 | 0.013 | 9 | 9 | 5 | 5 | 8 |
| GAB | 0.020 | 0.020 | 0.020 | 0.020 | 0.020 | 35 | 31 | 31 | 34 | 36 |
| GMB | 0.020 | 0.020 | 0.020 | 0.020 | 0.020 | 45 | 43 | 45 | 47 | 48 |
| GHA | 0.015 | 0.016 | 0.014 | 0.016 | 0.016 | 11 | 10 | 10 | 12 | 13 |
| GIN | 0.013 | 0.017 | 0.016 | 0.014 | 0.013 | 7 | 13 | 12 | 11 | 9 |
| KEN | 0.015 | 0.012 | 0.013 | 0.012 | 0.012 | 10 | 6 | 6 | 8 | 6 |
| LSO | 0.020 | 0.020 | 0.020 | 0.020 | 0.020 | 41 | 46 | 42 | 43 | 44 |
| LBR | 0.020 | 0.019 | 0.019 | 0.019 | 0.019 | 28 | 21 | 27 | 30 | 25 |
| MDG | 0.019 | 0.020 | 0.019 | 0.019 | 0.019 | 21 | 34 | 28 | 25 | 29 |
| MWI | 0.020 | 0.020 | 0.020 | 0.020 | 0.019 | 42 | 41 | 32 | 38 | 30 |
| MLI | 0.019 | 0.019 | 0.019 | 0.019 | 0.019 | 24 | 30 | 29 | 27 | 31 |
| MRT | 0.020 | 0.020 | 0.020 | 0.020 | 0.020 | 36 | 32 | 34 | 36 | 38 |
| MUS | 0.019 | 0.019 | 0.019 | 0.019 | 0.019 | 25 | 23 | 25 | 24 | 24 |
| MAR | 0.018 | 0.019 | 0.019 | 0.019 | 0.019 | 19 | 20 | 23 | 26 | 27 |
| MOZ | 0.018 | 0.017 | 0.017 | 0.017 | 0.017 | 18 | 12 | 14 | 16 | 18 |
| NAM | 0.020 | 0.019 | 0.019 | 0.019 | 0.020 | 30 | 29 | 30 | 31 | 34 |
| NER | 0.020 | 0.019 | 0.018 | 0.018 | 0.016 | 31 | 25 | 20 | 19 | 14 |
| NGA | 0.010 | 0.008 | 0.009 | 0.006 | 0.006 | 3 | 1 | 2 | 1 | 1 |
| RWA | 0.020 | 0.020 | 0.019 | 0.019 | 0.019 | 32 | 35 | 24 | 29 | 28 |
| SEN | 0.016 | 0.018 | 0.019 | 0.017 | 0.017 | 12 | 19 | 22 | 15 | 17 |
| SYC | 0.020 | 0.020 | 0.020 | 0.020 | 0.020 | 40 | 38 | 39 | 39 | 43 |
| SLE | 0.020 | 0.020 | 0.020 | 0.020 | 0.020 | 44 | 39 | 41 | 41 | 42 |
| ZAF | 0.005 | 0.009 | 0.011 | 0.011 | 0.012 | 1 | 3 | 3 | 6 | 7 |
| SSD | 0.019 | 0.020 | 0.020 | 0.020 | 0.020 | 23 | 40 | 37 | 32 | 39 |
| SDN | 0.019 | 0.018 | 0.018 | 0.018 | 0.018 | 20 | 18 | 19 | 22 | 22 |
| TZA | 0.018 | 0.016 | 0.016 | 0.014 | 0.014 | 13 | 11 | 11 | 10 | 11 |
| TGO | 0.020 | 0.020 | 0.020 | 0.020 | 0.020 | 33 | 33 | 36 | 37 | 37 |
| TUN | 0.020 | 0.020 | 0.020 | 0.020 | 0.020 | 39 | 36 | 40 | 40 | 40 |
| UGA | 0.018 | 0.018 | 0.017 | 0.016 | 0.017 | 14 | 14 | 13 | 13 | 15 |
| ZMB | 0.013 | 0.014 | 0.013 | 0.013 | 0.014 | 6 | 8 | 9 | 9 | 10 |
| ZWE | 0.018 | 0.018 | 0.017 | 0.017 | 0.017 | 16 | 15 | 15 | 17 | 19 |
