# Supplementary material for: Chinese influence in Africa by integrated regret theory and multi-criteria decision analysis
Source: PLoS One. 2025 Nov 12;20(11):e0336681. doi: 10.1371/journal.pone.0336681 (PMC12611122; doi:10.1371/journal.pone.0336681)
Supplement: Attachment — Submitted filename: Author response-revised.docx [file pone.0336681.s002.docx]

**RESPONSE LETTER**

Chinese influence in Africa by integrated regret theory and multi-criteria decision analysis

Dear Editor and reviewers,

Thank you for your valuable feedback provided regarding our manuscript. The authors greatly appreciate their thoughtful comments, as they have been instrumental in improving our paper and guiding our research. We have carefully reviewed the editor's and reviewers' suggestions and incorporated them into our revision. We hope to meet with your approval. Please kindly help us to see the following contents.

| Editor/Reviewer | Comments | Author response | Content revisions |
| --- | --- | --- | --- |
| Journal requirements | 1. Please ensure that your manuscript meets PLOS ONE's style requirements, including those for file naming. | The authors fully agree and have revised the manuscript in accordance with this requirement. | The revised manuscript format. |
|  | 2. We note that the grant information you provided in the ‘Funding Information’ and ‘Financial Disclosure’ sections do not match. When you resubmit, please ensure that you provide the correct grant numbers for the awards you received for your study in the ‘Funding Information’ section. | The corresponding author has updated the funding and financial disclosure. | Funding and financial disclosure |
|  | 3. Thank you for stating the following financial disclosure: This research is partial funded by University of Economics Ho Chi Minh City, VietNam. Please state what role the funders took in the study. If the funders had no role, please state: "The funders had no role in study design, data collection and analysis, decision to publish, or preparation of the manuscript." If this statement is not correct you must amend it as needed. Please include this amended Role of Funder statement in your cover letter; we will change the online submission form on your behalf. | The corresponding author has updated the funding as “This research is partial funded by University of Economics Ho Chi Minh City, Vietnam. The funders had no role in study design, data collection and analysis, decision to publish, or preparation of the manuscript." | Funding information |
|  | 4. We note that your Data Availability Statement is currently as follows: All relevant data are within the manuscript and its Supporting Information files Please confirm at this time whether or not your submission contains all raw data required to replicate the results of your study. Authors must share the “minimal data set” for their submission. | We have submitted the “minimal data set” of the study along with the revised manuscript. | The minimal data set was submitted as a supplementary file. |
|  | 5. We note that Figure 7 in your submission contain [map/satellite] images which may be copyrighted. All PLOS content is published under the Creative Commons Attribution License (CC BY 4.0), which means that the manuscript, images, and Supporting Information files will be freely available online, and any third party is permitted to access, download, copy, distribute, and use these materials in any way, even commercially, with proper attribution. For these reasons, we cannot publish previously copyrighted maps or satellite images created using proprietary data, such as Google software (Google Maps, Street View, and Earth). For more information, see our copyright guidelines: <http://journals.plos.org/plosone/s/licenses-and-copyright>.   We require you to either (1) present written permission from the copyright holder to publish these figures specifically under the CC BY 4.0 license, or (2) remove the figures from your submission | We used visualization tools to illustrate the research results. We replaced Figure 7 to ensure this requirement. | Figure 7 |
|  | 6. Please include a copy of Table A1-A4, A5-A8, A9-A12, A13-A22, A23-A27 and A28 which you refer to in your text on page 11, 12, 13, 16, and 18. | We have submitted the “minimal data set” of the study along with the revised manuscript. | The minimal data set was submitted as a supplementary file. |
| Reviewer #1 | The manuscript seeks to analyze Chinese influence in 49 African countries during 2018-22 through a combination of regret theory and decision-making approaches using economic indicators. The motivation is clear and commendable. But the application of regret theory or the choice of MCDM method lacks strong theoretical support. These models are typically used in behavioral decision-making contexts and there is no clear justification in the study why these methods are appropriate for testing the main hypothesis. The method seems forced and lacks empirical validation.  The paper presents an ambitious attempt to quantify Chinese influence in Africa, but it suffers from significant conceptual, methodological, and empirical weaknesses. The application of regret theory is not convincingly justified, the data is incomplete and unverified, and the chosen methodology lacks robustness in assessing geopolitical influence. The study does not significantly advance existing research on China-Africa relations, and its results are unlikely to provide useful insights for policymakers.  The key motivation of the paper suggests that with improvements in the technique, data and further refinement and redefinition of "influence" the paper could have potential. | Thanks for your comments. Please allow us to break-down your comments as several points and response point-to-point as below. |  |
|  | The application of regret theory or the choice of MCDM method lacks strong theoretical support. These models are typically used in behavioral decision-making contexts and there is no clear justification in the study why these methods are appropriate for testing the main hypothesis. | This concern is well understood, and in the revised manuscript, we have addressed it in both the literature review and the theoretical foundation (Theoretical Foundation). Regret theory is not applied to suggest emotional reasoning by states but rather to model bounded rationality and preference reversals in complex strategic environments. As numerous studies in behavioral economics and policy analysis have shown (e.g., Loomes & Sugden, 1982), decision-makers often seek to minimize anticipated regret, particularly under uncertainty. In the context of geopolitical alignment and development cooperation, African governments face multiple, often conflicting criteria (e.g., economic benefit, dependency risk, sovereignty concerns). The regret theory-based model is used to simulate this boundedly rational trade-off behavior.  MCDM was chosen precisely because it is suited to situations where decisions are made across multiple, heterogeneous criteria, which is inherently true of China-Africa economic relations. The combination of LOPCOW and MAIRCA offers a robust framework for analyzing objective weights and ideal-real comparisons, respectively. The conceptual diagram (Figure 3) clearly illustrate how each component contributes to modeling influence from a behavioral and data-driven perspective. | Theoretical Foundation section  Literature review section |
|  | The method seems forced and lacks empirical validation... the study does not significantly advance existing research. | The concept of “influence” has been more rigorously defined in **Defining Influence** section as a **multidimensional measure of economic embeddedness and strategic exposure**. Rather than framing influence purely as a political or coercive construct, our model views it as a composite function of capital flows, trade intensity, infrastructure dependency, labor presence, and financial leverage—all of which are well-documented mechanisms through which China engages African partners.  This refined definition is mapped explicitly to our six indicators in Justification of Indicators section. Furthermore, we have demonstrated how this quantitative approach **complements** existing qualitative literature by offering a replicable and structured framework for comparative assessment—something underrepresented in the China-Africa literature to date. The study thus contributes both **methodologically** (by integrating behavioral MCDM) and **empirically** (by producing a 5-year panel of influence rankings). | Defining Influence section  Justification of Indicators section |
|  | The data is incomplete and unverified. | We appreciate the concern. In the revised manuscript, we are now:   - Clearly list all data sources used, including SAIS China-Africa Research Initiative, IMF, ILO, WTO, and China’s National Bureau of Statistics. - Provide a supplementary file, which presents all data and computation results. | The supplementary file |
|  | The results are unlikely to provide useful insights for policymakers. | We respectfully disagree and have clarified the policy utility in the managerial implications section. The framework helps governments and development actors:   - Benchmark their strategic position relative to neighboring countries. - Identify which dimensions of engagement (e.g., trade vs. loans) dominate their relationship with China. - Anticipate potential imbalances or dependencies in specific sectors.   The regret-based model is particularly relevant in highlighting what countries might regret not pursuing—a valuable tool in strategic planning under uncertainty. This approach offers a forward-looking policy perspective that complements retrospective assessments in the literature. |  |
| Reviewer #2 | Lack of PLOS required formatting (page numbers, line numbers) made navigating and providing feedback on this manuscript difficult. Page numbers below refer to the reviewer copy. | The authors fully agree and have revised the manuscript in accordance with this requirement. | The revised manuscript formats with PLOS requirement. |
|  | The structure is in line with a monograph dissertation, rather than an article. While the authors should be commended on the thorough history and literature review of the methods, that component is much more in valuable for a scoping review article (which the authors should consider splitting off) than it is a for a hypothesis-driven article. Moreover, the studies that are extensively explained in the literature review do not make an appearance in the discussion section of the manuscript, so they do not aid in the author’s interpretation. | We appreciate this thoughtful comment and recognize that the manuscript initially adopted a structure more aligned with a methodological monograph. While our study is not a traditional hypothesis-testing article, it is **model-driven and exploratory**, designed to operationalize and apply an integrated regret theory-based MCDM framework in the context of China-Africa relations. We agree that the earlier literature review was extensive and have now revised it for focus and brevity, retaining only essential references that directly support the modeling framework.  To improve clarity and structure, a dedicated **“Theoretical Foundation”** subsection has been introduced within the **Introduction** section. This subsection briefly explains how regret theory, LOPCOW, and MAIRCA complement one another, and why their integration is conceptually suited to multi-dimensional, bounded-rationality-driven geopolitical analysis.  To further clarify the framework, **Table 1** has been added to concisely summarize each method’s role within the study: LOPCOW for weighting, MAIRCA for comparative ranking, and regret theory for capturing behavioral responses. This allows readers to understand each method’s function without being overwhelmed by methodological history. | Literature review section and introduction section.  Table 1 |
|  | The structure of the manuscript makes following the authors’ argument very difficult. Papers should be so logical that a reader should be able to guess your next steps. Unfortunately, the manuscript bounces between ideas that are not relevant to the study at hand. The methods section should be reordered (see comments below) to make clear what the authors are attempting.  The manuscript also needs to have separate and delineated results and discussion sections.  The methods also need to be | We appreciate this important structural critique and have addressed it through the following revisions:   - The **Introduction has been restructured** with four clearly labeled subsections:   - Motivation   - Defining influence   - Theoretical foundation   - Justification of indicators   - Research objective - The **Methodology section now begins with a brief overview paragraph** summarizing the full analytical process, followed by logically sequenced subsections detailing each step (weighting → regret adjustment → gap analysis). - The **Results and Discussion sections have been clearly separated**. The Results section focuses purely on quantitative outputs, while the Discussion interprets these outputs in the context of existing literature and policymaking.   These changes provide a clearer narrative thread, allowing the reader to follow the argument progressively from motivation to evidence to interpretation. | The introduction section  The brief overview paragraph of the methodology section  The results and discussion sections |
|  | The part of the paper that more or less lines up with a discussion section is underinterpreted, and needs to do more to help the reader understand the connection between the findings and the suggested policy implications. | This is a highly constructive point. The **Discussion section has been substantially expanded** and reframed around three core themes:   1. We now interpret the results of the influence rankings in light of regional variation, sectoral investment differences, and changes over time. 2. We explain how regret theory helps simulate real-world decision-making under uncertainty, particularly in contexts where African governments must navigate trade-offs between competing sources of foreign engagement. 3. A revised managerial implications subsection now articulates how governments and international stakeholders can:    - Benchmark their engagement strategies.    - Identify overexposure to particular channels of influence (e.g., loans vs. trade).    - Inform decisions on diversification, negotiation, and alignment with national development priorities.   This interpretive depth has been added specifically in response to the reviewer’s feedback and now better links empirical findings to actionable insights. | Discussion section  The policy implication section |
|  | Page 8: subsections in the introduction/lit review would be helpful to orient the reader. | Thank you for this comment. The introduction and literature review sections have been restructured in the revised manuscript. | Introduction and literature review |
|  | Page 9 top: what does “without political conditions” mean? When we’re talking about the political economy of nation-state engagement strategies, any agreement’s conditions (including “purely economic” ones) could be interpreted as political conditions. | Thank you for raising this important point. We agree that the phrase “without political conditions” can be overly simplistic in the context of international engagement, where even economic agreements inherently reflect political choices and priorities. Our intended meaning was to highlight the widely noted contrast in conditionality frameworks between Chinese and traditional Western development partners.  Specifically, Western aid and investment frameworks have historically tied financial assistance to political or governance-related conditions, such as democratization, human rights reforms, or transparency requirements. In contrast, Chinese engagements in Africa—particularly within the Belt and Road Initiative—are frequently described in policy and academic discourse as being structured around mutual economic benefit and non-interference, without overt requirements for political reform or institutional restructuring.  To avoid misunderstanding, we have revised the sentence to better reflect this distinction:  “China's approach is commonly framed as emphasizing economic cooperation and infrastructure investment without overt conditionality related to domestic political reforms—unlike traditional Western development models that often tie aid and loans to governance or policy change benchmarks. (10)”  This revised phrasing better acknowledges the political economy complexity of international agreements while accurately conveying the structural difference in conditionality practices. | Introduction section |
|  | Page 9 top: what kinds of concerns? | Allow us to respond to your question as follows:  These "concerns" were evaluated and analyzed in the study by C.Mlambo et al. <https://doi.org/10.1080/10971475.2016.1179023>  We summarize their findings as:  "concerns about dependency, sovereignty, and long-term development trajectories" |  |
|  | Page 9 mid: unclear “nuanced aspects beyond conventional...” | We have rewritten and clarified in the introduction section. |  |
|  | Page 9 mid: needs to rephrase “approach aims...”. You’re describing requirements for the approach in the paragraph (since you don’t describe the approach until the next paragraph), so language should reflect that. | Thanks for your comment.  The original sentence beginning with “This approach aims…” has been reworded throughout to instead refer to requirements for the analytical framework or the need for a model that can address complexity and behavioral variability. | The introduction section |
|  | Overall, the combination of these 4 theories is an interesting development and it makes sense to solve specific issues that you’re trying to model. However, currently, this is lost in the structure of the paragraphs. | A dedicated subsection titled **“Theoretical Foundation”** has been added to clearly introduce and explain how the four elements—MCDM, LOPCOW, MAIRCA, and Regret Theory—fit together and why they are appropriate for the study. | The introduction section |
|  | Page 9 bottom: MCDM, LOPCOW, MAIRCA, and Regret Theory come out of nowhere (you don’t explain until the next page). Need to motivate (with a subsection heading or one sentence) why you’re suddenly introducing this. | This problem has been fully resolved by:   - Adding subsection headers to guide readers through the logic of the introduction. - Preceding the methodological content with a paragraph that clearly motivates the need for an integrated framework and how each method contributes. - Including Table 1 to summarize roles, outputs, and relationships between methods. | The introduction section  Table 1 |
|  | 10: a figure illustrating how these theories are combined in a framework or a table would be really helpful to the reader. A table would allow you to more clearly compare and contrast what each theory adds to your framework, and would allow you to significantly reduce the amount of text in this section. | Thanks for your comment. We have been adding Table 1 to clarify the proposed framework. |  |
|  | 10: who is the actor that can have the anticipatory emotion of regret here? A nation-state is made up of people or be helmed by a person/a group of people, but does not inherently have emotions. Or unless you are describing a behavior (without ascribing emotion), which then should be clarified. | We appreciate the reviewer’s insightful observation. We fully agree that nation-states, as collective entities, do not possess emotions in a literal sense. Our application of regret theory does not imply that a state as a whole experiences emotional regret. Rather, the framework is used to simulate boundedly rational behavior by policymakers or governing institutions operating under uncertainty.  The concept of anticipatory regret is employed in this study as a behavioral modeling tool—not as a psychological attribution to the state, but as a representation of decision-making dynamics where the potential for future suboptimal outcomes influences current choices. This use is consistent with how regret theory has been applied in behavioral economics and policy analysis, where institutional actors are understood to evaluate scenarios based on perceived losses or missed opportunities.  Thus, regret in this context is understood as a heuristic for strategic trade-off consideration, especially in complex geopolitical settings where nations weigh multiple criteria under constraint. We have aimed to apply this behavioral logic carefully and metaphorically, and we appreciate the opportunity to clarify this distinction. |  |
|  | Page 10: the three paragraphs here in the middle could be condensed to a single paragraph due to redundant writing. Novelty of approach should be moved to the discussion. | Thanks for your comment. The introduction section has been restructured to improve logical flow and readability. The novelty section has been moved to the discussion section. | The introduction section.  The discussion. |
|  | Page 11: Given Table 1, the literature review in this format seems redundant. One possible solution is to cut the models that each other author has used from the text (since it’s communicated more effectively in the table). You should also try to summarize more and guide the reader to where exactly the gap in the literature is (as you do top of page 12). The remainder of the paragraph at the bottom of page 11 that starts “In 2023, several studies…” does this rather effectively. Another solution is to cut the entire paragraph in page 11 (again because Table 1 exists) and condense the citations into the short paragraph at the top of page 12. Alternatively, you could maybe summarize the contents of page 11 in 2-5 short sentences before jumping into the paragraph on 12. | We thank the reviewer for this helpful observation. We agree that the original version of the literature review was overly detailed in its explanation of each study’s methodology, which created redundancy given the presence of Table 1.  In the revised manuscript, this subsection has been **significantly condensed** and restructured to focus on the **substantive insights** and thematic contributions of the reviewed studies, while all methodological details are now retained **only in Table 1** for clarity and efficiency. We have followed the reviewer’s suggestion to summarize the reviewed literature in a concise narrative and then **guide the reader directly to the existing research gap**—namely, the lack of decision-making frameworks that capture multi-criteria trade-offs and psychological factors like regret in China–Africa relations.  Additionally, the paragraph beginning “In 2023, several studies…” has been retained and revised to effectively frame recent thematic expansions in the literature providing a useful transition into our proposed methodological innovation.  We believe these changes have improved the focus and flow of the literature review, and we thank the reviewer again for this constructive recommendation. | The literature review section |
|  | Page 12, top: this is a very effective paragraph. | Thank you for this comment. |  |
|  | Page 12, section 2.2: the entire history of and literature review of MCDM is not germane to your paper (unless you wanted to split off a scoping review paper as a second submission). The only part that is germane is the section starting with “Bounded rationality”, although this can also be dramatically condensed and summarized on how it relates specifically to MAIRCA and Regret Theory. This could then lead into the paragraph that starts near the top of page 13. | Thank you for this comment. We thank the reviewer for this valuable suggestion. In response, we have removed the historical account of MCDM development and condensed the literature review to focus on those components most relevant to our methodological integration—particularly bounded rationality, regret-based behavior, and their alignment with MAIRCA and LOPCOW techniques. The revised subsection now directly links recent behavioral MCDM studies to the analytical tools used in this paper, while removing any background detail that may distract from the core contribution. | Literature review |
|  | Page 13 can be condensed given Table 2. | We agree that the earlier paragraph-by-paragraph explanation of each study duplicated information already present in Table 2. In the revised manuscript, the **textual narrative has been streamlined**, and **Table 2 is now the primary source** for summarizing methodological advances in recent behavioral MCDM literature. The paragraph serves to synthesize key themes from the table and provide a transition into the justification for our integrated framework, eliminating redundancy while preserving clarity. | Literature review |
|  | Page 14: your paper would greatly benefit from an objective statement since it’s not clear at the top of page 14 what exactly is it that you’re trying to study (and thus difficult for the reader to evaluate the merits of the methodology). | We have added a clear and specific objective statement at the beginning of Objective and Overview of Method section that outlines the purpose and structure of the methodology, explaining what the model seeks to measure (Chinese influence) and how it does so (through regret-based MCDM with LOPCOW and MAIRCA). | The methodology section |
|  | Page 14 middle: you need to include a short sentence or paragraph explaining the general method you will employ. | A concise summary paragraph has been inserted at the beginning of the methodology section, explaining the general steps: normalization, behavioral utility modeling, objective weighting, and gap-based ranking. | Literature review |
|  | Page 14 middle: would be useful in that general method paragraph to mention that you are conducting a gap analysis (this doesn’t really become clear despite a brief mention in figure 3 until page 17). | The revised summary paragraph now explicitly states that a gap analysis is central to the methodology, used to measure the divergence between theoretical and observed country performance. | The methodology section |
|  | Page 14 middle: why is the explanation of regret theory literature in the methods section and not the literature review section like for the other methods? Also, this can be condensed as the reader could always go read the former paper. Section 3.1 should be cut, and section 3.2 (which I’m assuming is the authors’ novel contribution) should be the part to explain the regret theory method. | We have moved the theoretical explanation of regret theory—including its origin, mathematical formalization, and psychological basis—to the literature review section. The methodology section now focuses exclusively on the computational implementation of regret-adjusted utility. | The methodology section |
|  | Page 17 top: Excluded countries need to be listed (fine if it’s in an appendix). |  |  |
|  | Page 17: Some background on the indicators should have been a subsection of your introduction. The reader has merely the authors’ assertion that these are vital indicators. They seem on the face like reasonable indicators, but that needs to be established in the background. | We have provided the subsection “Justification of indicators” in the introduction. | The introduction section |
|  | Page 19 bottom: potential reasons for FDI stock variability should have been established in the background, or, if it is a finding that is novel to your study, the potential reasons should be discussed in the discussion, not the results. | We have restructured the case study (results) section and discussion sections. | The case study section  The discussion section |
|  | In general pages 19-22: discussion points and should not be in results, but in discussion. | We have restructured the case study (results) section and discussion sections. | The case study section  The discussion section |
|  | Page 17-19: indicators and methods should be in the methods section, not the results section. | We appreciate the reviewer’s comment. While the general methodology is detailed in the Methodology section, the indicators used (C1–C6) are specific to this case study and form a contextual component of the analysis, not the method itself. Therefore, their empirical values, observed variability, and role in the outcome are appropriately introduced and analyzed within the Results section. The method remains generalizable, but the operationalization of variables must be case-specific. |  |
|  | Figure 8: Unclear what figure 8 displays. | The rankings provided for African countries from 2018 to 2022, in relation to Chinese influence, display interesting trends and shifts over the five-year period as shown in Fig 8. |  |
|  | Overall, the manuscript is too wordy. There are sentence simplifications and some condensing of paragraphs that could easily happen that would improve overall the ability of the reader to follow your argument. | We have rewritten and condensed the entire manuscript. Number of words reduced from 11,873 to 9761 words. | The entire manuscript. |
|  | Page 19 mid: Needs either “the Chinese” or “Chinese investments” | We have updated the policy implementation section | The discussion section |
|  | Page 20: typo in Figure 4: “standard deviation” | We corrected it. | Figure 4 |
