# Supplementary material for: Chinese influence in Africa by integrated regret theory and multi-criteria decision analysis
Source: PLoS One. 2025 Nov 12;20(11):e0336681. doi: 10.1371/journal.pone.0336681 (PMC12611122; doi:10.1371/journal.pone.0336681)
Supplement: Attachment — Submitted filename: Author response-revised-2.docx [file pone.0336681.s003.docx]

**RESPONSE LETTER**

Chinese influence in Africa by integrated regret theory and multi-criteria decision analysis

Dear Editor and reviewers,

Thank you for your valuable feedback provided regarding our manuscript. The authors greatly appreciate their thoughtful comments, as they have been instrumental in improving our paper and guiding our research. We have carefully reviewed the editor's and reviewers' suggestions and incorporated them into our revision. We hope to meet with your approval. Please kindly help us to see the following contents.

| Editor/Reviewer | Comments | Author response | Content revisions |
| --- | --- | --- | --- |
| Journal requirements | Upon internal evaluation of the reviews provided, we kindly request you to disregard the reviewer report provided by Reviewer 3. No amendments are required in response to reviewer 3’s comments. | Well noted with thanks. |  |
|  | I commend you on the substantial improvements made to your manuscript, particularly in terms of structure, clarity, and overall readability. Both reviewers have acknowledged these enhancements. However, several substantive issues remain that need to be addressed before the manuscript can be considered for publication. Therefore, I am recommending a major revision at this stage.  Reviewer 1 has noted that the manuscript still lacks a well-developed limitations subsection. The current paragraph at the end of the conclusion should be moved to the discussion section and significantly expanded. Additionally, a number of background statements are presented without citations, please ensure that all such claims are properly supported by references. Reviewer 1 also offers helpful technical suggestions, including improvements to figures and tables (e.g., clarifying legends and removing redundant content from Table 2). | Thank you very much for your feedback. We have addressed each point according to reviewer 1's (reviewer #2 in the decision letter) comments.  However, we disregarded the comments of reviewer 2 (reviewer #3 in the decision letter) as instructed by the editorial office above. | The revised manuscript |
| Reviewer 1  (Reviewer #2) | The manuscript is missing a limitations subsection in the discussion (the single paragraph at the end of the conclusion should be moved and expanded). | Thank you for pointing this out. In response, a dedicated Limitations subsection has been added to the Discussion section. The previously brief paragraph from the conclusion has been relocated and substantially expanded to provide a more comprehensive account of the study’s constraints and opportunities for future research. |  |
|  | 62: needs a citation  67: needs a citation  70: needs a citation | We thank the reviewer for this valuable feedback. The revised manuscript now includes appropriate citations to support the conceptual framing of influence and its asymmetrical and regionally differentiated nature | Citations updated |
|  | 100-107: these would benefit from citations showing they’ve been used or thought of in this way in the past. | We thank the reviewer for this helpful suggestion. The manuscript has been revised to include appropriate citations that support the use of each of the six indicators as meaningful dimensions of Chinese influence in Africa. | Citations updated |
|  | 111: are there studies that have relied only on trade/investment as a proxy for influence? Good to cite a few here (or better yet cite a study that explains this as a limitation of previous studies) | Thank you for this insightful comment. We have revised the statement to include recent studies that rely solely on trade or investment as proxies for influence, as well as studies that critique this narrow approach. This strengthens the justification for the multidimensional framework proposed in our study. | Line 111-116 |
|  | Table 2: by my read, the role of table 2 is to show current state of literature which helps justify your study. The last row with your study is unnecessary for that purpose and should be removed since you already address it well in 154-156. | We thank the reviewer for this helpful observation. The final row referring to our study has been removed from Table 2, in line with the purpose of the table to review the current literature landscape. As suggested, the study’s own methodological contribution remains appropriately described in lines 154–156 of the manuscript. | Table 2 |
|  | Tables 6-9: Inclusion of legends would be helpful mostly to orient the reader towards what the colors and shading mean | We appreciate the reviewer’s attention to clarity in data presentation. To avoid any potential confusion, the color shading originally used to highlight value differences in Tables 6–9 has now been removed. The primary purpose of the shading was to draw visual attention to numerical differences, but we agree that simplified formatting enhances readability without requiring additional legends. | Table 6-9 |
|  | Figure 7: Repeated colors (and similar colors) make it difficult to tell which countries are which. Consider putting the 3-letter country code as a column on the left side next to each country’s starting position. | We thank the reviewer for this helpful observation. It appears that the comment may be referring to Figure 8 rather than Figure 7, as Figure 8 presents the five-year ranking trends for each country. Due to the high number of alternatives (49 countries), repeated or similar colors are indeed unavoidable, which we recognize may affect readability. To improve clarity and resolve this issue, the visual plot (Figure 8) has been replaced with a more accessible format—Table 10, which presents the same year-by-year ranking data in a structured tabular layout using 3-letter country codes. This allows readers to follow changes in rank over time without relying on color differentiation. | Change Figure 8 to Table 10 |
|  | 411, 413: refer to the citations: which earlier findings? | Thank you for highlighting the need for greater clarity regarding the referenced findings. We have revised the text to explicitly refer to the specific studies and findings being cited, thereby improving the transparency and traceability of the claims. | Line 411-415 |
|  | 427-428: needs a citation | Thank you for pointing this out. A supporting citation has been added to contextualize the observed increase in Chinese labor presence and its implications. This inclusion links the empirical trend to documented shifts in China’s operational strategies in Africa. | Line 424-427 |
|  | 430-432: is there anything you can do to corroborate this? At minimum a citation. | Thank you for your suggestion. A citation has been added to substantiate the claim regarding the strategic importance of these nations in China's Africa engagement. This strengthens the argument by referencing established literature on China's geopolitical and economic priorities on the continent. | Line 430-432 |
|  | 435: needs a citation  436: needs a citation | Thank you for your valuable feedback. A citation has been added to support the statement regarding the limited strategic importance of certain African countries in China’s broader foreign policy approach. This helps contextualize the observed lower levels of engagement. | Line 435-436 |
|  | 437-438: needs a citation | Thank you for your observation. A citation has now been added to support the claim regarding Nigeria’s strategic relationship with China and its sectoral engagement, particularly in oil, transport, and digital infrastructure. This reinforces the interpretation of the ranking trends provided in Table 10. | Line 437-438 |
|  | 100: expand since 1st appearance of acronym FDI in the text (though it shows up earlier in a figure) | We appreciate the reviewer’s attention to clarity. The acronym FDI has now been expanded to Foreign Direct Investment at its first appearance in the main text to ensure accessibility for all readers. Although the abbreviation appeared earlier in a figure, it is now fully defined in the narrative for consistency and reader comprehension. | Line 100 |
