# Supplementary material for: Chinese influence in Africa by integrated regret theory and multi-criteria decision analysis
Source: PLoS One. 2025 Nov 12;20(11):e0336681. doi: 10.1371/journal.pone.0336681 (PMC12611122; doi:10.1371/journal.pone.0336681)
Supplement: Attachment — Submitted filename: PONE-D-25-05084R2.docx [file pone.0336681.s004.docx]

For the Manuscript Number (**PONE-D-25-05084R2**), Title “Chinese Influence in Africa by Integrated Regret Theory and Multi-criteria Decision Analysis”, I suggest being accepted based on the following points:

1- Authors have revised and updated the introduction and literature properly.
2- The effort is clearly beneficial to the community.
3- The manuscript has also precision and creative thinking.
4- The quality of work that was put into researching and presenting the methodology is commendable.
5- There have been several improvements made to the results analysis's level of detail.
