# Supplementary material for: Chinese influence in Africa by integrated regret theory and multi-criteria decision analysis
Source: PLoS One. 2025 Nov 12;20(11):e0336681. doi: 10.1371/journal.pone.0336681 (PMC12611122; doi:10.1371/journal.pone.0336681)
Supplement: Attachment — Submitted filename: Author response-revised-3.docx [file pone.0336681.s005.docx]

**RESPONSE LETTER**

Chinese influence in Africa by integrated regret theory and multi-criteria decision analysis

Dear Editor and reviewers,

Thank you for your valuable feedback provided regarding our manuscript. The authors greatly appreciate their thoughtful comments, as they have been instrumental in improving our paper and guiding our research. We have carefully reviewed the editor's and reviewers' suggestions and incorporated them into our revision. We hope to meet with your approval. Please kindly help us to see the following contents.

| Editor/Reviewer | Comments | Author response | Content revisions |
| --- | --- | --- | --- |
| Journal requirements | Upon internal evaluation of the reviews provided, we kindly request you to disregard the reviewer report provided by Reviewer 5. No amendments are required in response to reviewer 5’s comments. | Well noted with thanks. |  |
| Reviewer 1  (Reviewer #5) | Although the paper explicitly treats “influence” as a multidimensional construct, the theoretical framing would benefit from greater clarity through stronger connections to contemporary debates in international political economy. In addition to economic embeddedness, the dimensions of institutional trust and asymmetrical interdependencies should be incorporated, as these factors are instrumental in explaining the persistence of Chinese influence. | We respectfully disagree with the reviewer’s comment. The dimensions of institutional trust and asymmetrical interdependencies are already inherently embedded within the theoretical framework and indicator selection of the study. Specifically, institutional trust is reflected through the inclusion of loan-based financial relations and construction project revenues, which require sustained bilateral confidence and contractual reliability between China and African states. Likewise, asymmetrical interdependencies are captured through trade flows, FDI stock, and labor deployment, which collectively represent structural imbalances and long-term dependency mechanisms central to international political economy debates. Therefore, while the manuscript may not explicitly label these dimensions, they are analytically integrated within both the conceptual framing of “influence” and the empirical evaluation through the regret theory-based MCDM model. | None |
|  | The study could be further enriched by situating Chinese influence within comparative state structures. For instance, research on hierarchical governance demonstrates its role in shaping both intercity and international linkages. Incorporating evidence from studies such as “The Innovation Effect of Administrative Hierarchy on Intercity Connection” would strengthen the analysis by showing how administrative arrangements mediate influence dynamics. | We appreciate the reviewer’s insightful suggestion. However, the current study’s primary objective is to develop and apply a regret theory-based multi-criteria decision-making (MCDM) framework to quantify and analyze Chinese influence in Africa through economic and behavioral dimensions rather than to conduct a comparative analysis across different state structures. While hierarchical governance and administrative arrangements indeed play an important role in shaping intercity and international linkages, such factors extend beyond the empirical and methodological scope of this paper, which focuses on measurable economic indicators and regret-adjusted behavioral modeling. Nonetheless, the reviewer’s point is well taken, and comparative hierarchical governance perspectives are acknowledged as a valuable direction for future research that could complement the present model by examining how administrative structures mediate or amplify the persistence of external influence. | None |
|  | Given the framework’s integration of regret theory and bounded rationality, the link between trust (or mistrust) among actors and their perceptions of regret and opportunity costs should be made more explicit. Reference to studies such as Jiang, Zhao, Dong, & Hu (2024) doi: <https://doi.org/10.1016/j.inffus.2023.102173> would enhance the behavioral dimension of the model by embedding trust within decision-making frameworks. | We appreciate the reviewer’s thoughtful observation. However, the study’s integration of regret theory and bounded rationality already implicitly accounts for the role of trust and mistrust through the behavioral constructs of anticipated regret and opportunity cost perception. In the proposed framework, decision-makers are modeled as boundedly rational actors who assess engagement outcomes under uncertainty, factoring in both expected benefits and the potential regret associated with suboptimal choices. These behavioral responses inherently capture the influence of trust dynamics—since higher institutional or inter-actor trust reduces perceived regret and opportunity costs, whereas mistrust amplifies them. Therefore, while the manuscript does not explicitly reference the trust–regret linkage as in Jiang, Zhao, Dong, & Hu (2024), the underlying mechanism is conceptually embedded within the regret utility formulation and behavioral interpretation of the model. | None |
|  | While addressing Chinese labor flows into Africa, the manuscript overlooks the regulatory and governance frameworks shaping such dynamics. A deeper analysis of how labor law regimes condition socio-economic outcomes would add value. Incorporating works such as “The Effect of Changing Criminal Laws on the Regulation of Labor Conflicts in P.R. China” would provide the necessary legal context. | We thank the reviewer for this valuable comment. However, the focus of the present study is on quantifying Chinese influence in Africa through an integrated regret theory-based MCDM framework rather than on analyzing the legal or regulatory mechanisms underlying labor governance. The indicator of Chinese labor deployment (C4) is incorporated as an empirical measure of operational presence and economic influence, capturing the behavioral and structural dimensions of engagement rather than the juridical context in which such labor flows occur. While we recognize that labor law regimes—both in China and in host African countries—undoubtedly shape these dynamics, incorporating a comparative legal analysis would extend beyond the methodological and empirical scope of this work. Nevertheless, we agree that future studies could profitably build on the current framework by integrating regulatory and governance dimensions to explore how legal structures mediate the socio-economic consequences of Chinese labor movements in Africa. | None |
|  | The analysis does not sufficiently engage with the role of African human capital in shaping reciprocal relations with China. Expanding the discussion to consider labor mobility, graduate employment, and structural dependencies would be beneficial. For instance, Zhao, Chang, & Yusof (2023) offer useful insights in “Analysis of Job Search Success Factors among Chinese University Graduates A Pathway towards Employment-oriented Linkages.” | We appreciate the reviewer’s constructive observation. However, the current study’s analytical focus is directed toward evaluating Chinese influence in Africa through measurable economic and behavioral indicators within a regret theory-based MCDM framework, rather than examining reciprocal dynamics driven by African human capital formation or labor market interactions. While we acknowledge that African labor mobility, graduate employment, and capacity development play crucial roles in shaping the long-term reciprocity of China–Africa relations, these aspects fall outside the primary scope of the present quantitative model, which concentrates on the structural asymmetries of Chinese engagement. Nonetheless, we agree that future research could extend this framework by incorporating human capital indicators and employment-related metrics to explore how local labor structures and skill development mediate the balance of influence and dependency between China and African countries. | None |
|  | By focusing primarily on FDI, loans, and infrastructure, the paper underplays global supply chain vulnerabilities and their implications for African economies. Including a resilience-oriented perspective such as risks of supply disruptions and financial contagion would provide a more comprehensive assessment. Relevant methodological insights can be drawn ) on risk-based MCDM approaches, :Risk Identification and Prioritization in China's New Energy Vehicle Supply Chain: An Integrated Tanimoto Similarity and Fuzzy-DEMATEL Approach). | We appreciate the reviewer’s insightful suggestion. Nonetheless, the primary objective of this study is to assess Chinese influence in Africa through an integrated regret theory-based MCDM framework emphasizing economic engagement indicators such as FDI, loans, trade, labor, and construction revenues. The inclusion of global supply chain vulnerabilities and resilience dimensions, while undoubtedly important, extends beyond the study’s intended analytical scope, which is centered on quantifying influence rather than evaluating systemic risk or contagion pathways. However, we acknowledge the relevance of resilience-oriented approaches—such as those employing fuzzy-DEMATEL or other risk-based MCDM models—for future extensions of this research. These methods could enrich the current framework by incorporating interdependence risks, supply disruption factors, and contagion dynamics to provide a more comprehensive understanding of the stability and resilience of China–Africa economic linkages. | None |
|  | The integration of regret theory is innovative; however, the choice of parameters (e.g., α = 0.88, β = 0.3) lacks sufficient justification. The authors should clarify whether these were derived from prior literature, expert elicitation, or sensitivity analysis. Testing robustness under alternative parameter values would further strengthen confidence in the findings. | We appreciate the reviewer’s careful attention to the parameterization of the regret theory component. The chosen parameters (α = 0.88, β = 0.3) were not arbitrarily selected but are grounded in established behavioral decision-making literature, specifically following the empirical calibration proposed by Tversky and Kahneman (1992) in *Advances in Prospect Theory: Cumulative Representation of Uncertainty*. These parameter values have been widely adopted in subsequent studies employing regret or prospect-based decision models to represent realistic levels of risk and regret aversion among boundedly rational decision-makers. As stated in the manuscript, these values were applied to ensure methodological consistency with the foundational behavioral framework and to maintain comparability with prior research. Therefore, the parameters are theoretically justified rather than elicited or estimated through new sensitivity analysis within this study’s scope. | None |
|  | To ensure robustness and mitigate method-specific bias, the presented rankings should be validated using additional MCDM techniques such as TOPSIS, VIKOR, or fuzzy-DEMATEL, refer to doi: <https://doi.org/10.1016/j.ijme.2024.101018>.; doi: 10.15837/ijccc.2022.6.5010., doi: <https://doi.org/10.1016/j.solener.2024.112692>. This triangulation would demonstrate that the results are not overly sensitive to the selected model. | We appreciate the reviewer’s valuable recommendation regarding methodological validation. However, the intent of this study is not to perform a comparative analysis across multiple MCDM techniques but to introduce and demonstrate the applicability of an integrated regret theory-based LOPCOW–MAIRCA framework as a novel behavioral decision-making approach. The study’s objective is to highlight how regret-adjusted utilities and gap-based evaluations can capture bounded rationality in geopolitical contexts, rather than to test convergence across alternative MCDM algorithms. While we acknowledge that triangulation using methods such as TOPSIS, VIKOR, or fuzzy-DEMATEL could further support robustness testing, such validation would expand the study’s scope beyond its primary methodological innovation. Nonetheless, future research may incorporate these comparative methods to confirm stability and examine potential sensitivity variations across behavioral and non-behavioral MCDM frameworks. | None |
|  | The study is heavily structural and primarily quantitative, with limited incorporation of qualitative evidence. Triangulating the quantitative findings with case studies from contexts such as Kenya, Nigeria, or Ethiopia would enhance interpretability and increase policy relevance. | We thank the reviewer for this thoughtful suggestion. However, the design of this study is intentionally structured around a quantitative behavioral decision-making framework to ensure methodological consistency and objectivity in measuring Chinese influence across 49 African countries. The integration of qualitative evidence or case studies, while indeed valuable for contextual interpretation, would introduce heterogeneity that may compromise the comparability and statistical coherence of the regret theory-based MCDM model. The study’s primary aim is to establish a replicable quantitative framework capable of ranking and analyzing influence dynamics through objectively derived indicators. Nonetheless, we fully agree that country-level case studies—particularly from Kenya, Nigeria, or Ethiopia—could serve as meaningful extensions in future research to triangulate and contextualize the empirical results, thereby enhancing the interpretability and policy relevance of the model’s findings. | None |
|  | The policy recommendations are currently broad. It would be valuable to distinguish between immediate operational options (loan conditions, export market diversification) and long-term strategic imperatives (institutional capacity-building, governance reform). This would yield more actionable guidance for policymakers. | We appreciate the reviewer’s constructive feedback. However, the policy recommendations in the current version were intentionally presented at a broader strategic level to align with the study’s macro-analytical scope, which evaluates Chinese influence across 49 African countries rather than focusing on country-specific policy prescriptions. The objective was to provide a generalized framework applicable to a wide range of national contexts, emphasizing structural alignment, behavioral adaptation, and strategic balance in engagements with China. While the distinction between short-term operational measures (such as loan conditions and export diversification) and long-term strategic imperatives (such as institutional strengthening and governance reform) is indeed important, incorporating such granularity would require context-specific policy environments that extend beyond the current study’s comparative, quantitative design. Nevertheless, we agree that future extensions of this research could develop a two-tier policy framework distinguishing between immediate operational adjustments and long-term strategic reforms to enhance the practical applicability of the findings for individual policymakers. | None |
|  | Several aspects of presentation require improvement: (a) equations should be linked to illustrative examples and explained intuitively, (b) figures and tables should include more detailed captions with explicit reference to policy relevance, and (c) the discussion should more clearly integrate theoretical contributions with empirical findings to delineate the study’s novelty. | We thank the reviewer for these detailed presentation-related observations. However, the equations, figures, and tables in the current version were deliberately designed to maintain analytical clarity and methodological focus rather than to serve illustrative or pedagogical purposes. Each equation is accompanied by descriptive text explaining its computational role within the regret theory-based MCDM framework, ensuring methodological transparency consistent with academic standards for quantitative modeling. Similarly, the figures and tables are primarily intended to display empirical outcomes, such as weight distributions, ranking trends, and total gap analyses, rather than to provide policy narratives, which are instead addressed comprehensively in the discussion and policy implication sections.  Regarding the integration of theoretical and empirical components, the study’s novelty has been articulated through the behavioral extension of MCDM using regret theory to capture bounded rationality in geopolitical analysis. This conceptual advancement is directly reflected in the empirical findings through the interpretation of criteria weights and influence rankings. Therefore, while additional descriptive elaboration could further enhance accessibility, the current presentation format was intentionally maintained to preserve methodological precision and academic conciseness. | None |
